# PIK3CA in Cancer: Structure, Biology, Alterations, and Actionability

**DOI:** 10.3390/cancers18172798

**Published:** 2026-08-28

**Authors:** Alessandro Ottaiano, Carmine Picone, Mariachiara Santorsola, Massimiliano Berretta, Carmen Cutolo, Andrea Belli, Francesco Izzo, Nadia Di Carluccio, Aniello Acampora, Luca Tarotto, Salvatore Stilo, Marco Correra, Maurizio Capuozzo, Anna Chiara Carratù, Michele Caraglia, Guglielmo Nasti, Giovanni Savarese

**Affiliations:** 1 Istituto Nazionale Tumori di Napoli, IRCCS “G. Pascale”, Via M. Semmola, 80131 Naples, Italy; mariachiara.santorsola@istitutotumori.na.it (M.S.); carmen.cutolo@istitutotumori.na.it (C.C.); a.belli@istitutotumori.na.it (A.B.); f.izzo@istitutotumori.na.it (F.I.); nelloacampora19@gmail.com (A.A.); luca.tarotto@istitutotumori.na.it (L.T.); salvatore.stilo@istitutotumori.na.it (S.S.); m.correra@istitutotumori.na.it (M.C.); annachiara.carratu@istitutotumori.na.it (A.C.C.); g.nasti@istitutotumori.na.it (G.N.); 2 Department of Medicine and Health Science, “Vincenzo Tiberio” University of Molise, 86100 Campobasso, Italy; carmine.picone@unibas.it; 3 Department of Clinical and Experimental Medicine, University of Messina, 98122 Messina, Italy; mberretta@unime.it; 4 Department of Economics, University of Foggia, 71121 Foggia, Italy; nadia.dicarluccio@unifg.it; 5 Pharmaceutical Department, Asl Napoli 3 Sud, Via Marittima 3, 80056 Ercolano, Italy; 6 Department of Precision Medicine, University of Campania “Luigi Vanvitelli”, Via L. De Crecchio 7, 80138 Naples, Italy; michele.caraglia@unicampania.it; 7 Centro AMES, Via Padre Carmine Fico 24, 80013 Casalnuovo di Napoli, Italy; giovanni.savarese@centroames.it

**Keywords:** PIK3CA, PI3K signaling, precision oncology, targeted therapy

## Abstract

*PIK3CA*, which encodes the p110α catalytic subunit of phosphoinositide 3-kinase (PI3K), is one of the most frequently altered oncogenes in human cancer and a major driver of tumor initiation, progression, metastasis, and therapeutic resistance. Over the past two decades, advances in structural biology, cancer genomics, and translational research have substantially expanded our understanding of PIK3CA function and established the PI3K pathway as a clinically actionable therapeutic target. This review provides an overview of the structural organization and physiological functions of the PI3Kα complex, the molecular mechanisms underlying oncogenic activation, and the diverse spectrum of *PIK3CA* alterations across human malignancies. We also summarize the current landscape of PI3K-targeted therapies, highlighting both approved agents and emerging therapeutic strategies. Clinical evidence supports the rational integration of PI3K inhibitors with endocrine therapy, CDK4/6 inhibitors, MAPK pathway inhibitors, dual PI3K/mTOR inhibition, and immune checkpoint blockade. In addition, accumulating evidence indicates that PIK3CA plays a pivotal role in shaping the tumor immune microenvironment, providing a biological rationale for combining PI3K inhibition with immunotherapy. Finally, we discuss future directions in precision oncology, emphasizing integrated molecular profiling, liquid biopsy, single-cell and spatial technologies, functional genomics, and evolutionary approaches as complementary strategies to refine patient selection, overcome therapeutic resistance, and optimize clinical outcomes.

## Simple Summary

*PIK3CA* is one of the most frequently altered cancer-related genes and has become an important focus of precision oncology. However, the biological and clinical implications of *PIK3CA* alterations vary considerably across tumor types, and translating this knowledge into effective treatments remains challenging. This review provides a comprehensive, updated, and comparative overview of PIK3CA biology, its molecular alterations across human cancers, their prognostic and therapeutic relevance, and the current development of treatments targeting the pathway activated by PIK3CA. We critically examine both established evidence and remaining limitations, including treatment toxicity and resistance, and discuss emerging therapeutic and molecular approaches. By bringing these different aspects together, we aim to provide oncologists and researchers with a practical framework for understanding the current state of the field, interpreting available evidence, identifying unresolved questions, and designing future clinical and translational studies.

## 1. Introduction

Phosphatidylinositol-4,5-bisphosphate 3-kinase catalytic subunit alpha (*PIK3CA*) encodes the p110α catalytic subunit of class I phosphoinositide 3-kinases (PI3Ks), which play a central role in plasma membrane receptor-initiated signaling networks governing growth, proliferation, survival, metabolism, and angiogenesis [1]. Among the numerous genetic alterations implicated in human malignancies, mutations in *PIK3CA* rank among the most frequent, being detected across a wide range of tumor types including breast, colorectal, endometrial, cervical, and head and neck squamous cell carcinomas [1,2]. These mutations often confer a gain-of-function phenotype, promoting constitutive activation of the PI3K/protein kinase B (AKT)/mechanistic target of rapamycin (mTOR) pathway signaling cascade and thereby facilitating oncogenesis [2,3].

Over the past two decades, a growing body of evidence has underscored the significance of *PIK3CA* not only as a driver of tumorigenesis but also as a clinically actionable biomarker [4,5]. The identification of recurrent hotspot mutations, such as E542K, E545K, and H1047R, has catalyzed the development of targeted inhibitors and informed patient selection strategies in precision oncology [2,3,6]. Furthermore, comprehensive genomic profiling from consortia like The Cancer Genome Atlas (TCGA) and the International Cancer Genome Consortium (ICGC) has delineated the mutational landscape of *PIK3CA* and its co-alteration patterns, shedding light on both tumor-specific dependencies and resistance mechanisms [1,2].

In this review, we present a synthesis of the structural organization and biological functions of the PI3K complex, with a particular focus on the p110α subunit. We examine the prevalence and functional consequences of *PIK3CA* alterations in diverse cancer types, exploring how these mutations confer selective growth advantages and influence tumor behavior. We also discuss the spectrum of *PIK3CA* genetic aberrations, from canonical hotspots to rare variants, and their implications for therapy. Finally, we provide a critical appraisal of current and emerging therapeutic strategies targeting PIK3CA, including data from clinical trials evaluating the efficacy of isoform-specific inhibitors, and outline future directions for optimizing treatment of PIK3CA-driven malignancies.

## 2. Structure of PIK3CA

**Figure 1:**
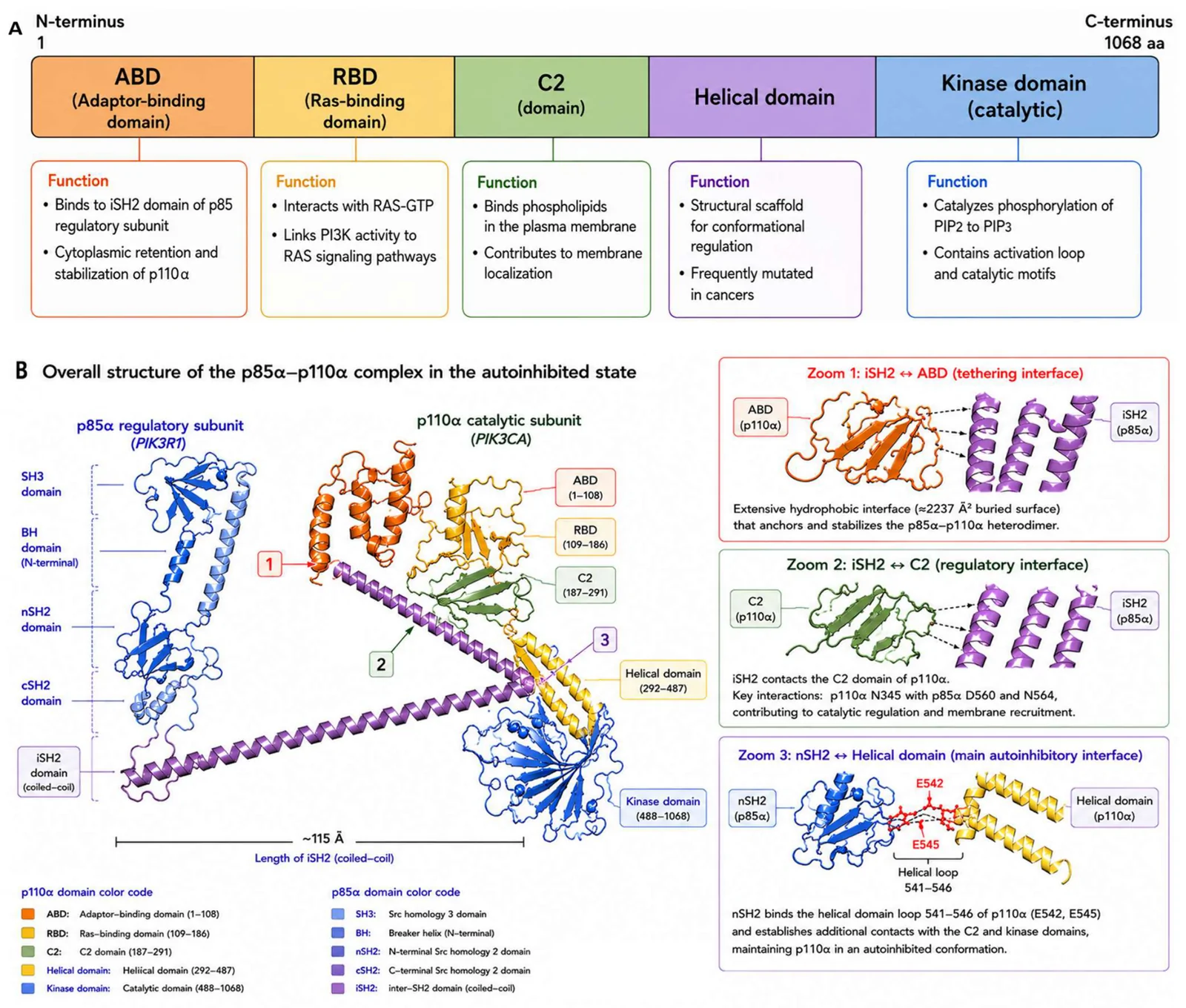
Structural organization of the Class IA phosphoinositide 3-kinase (PI3K) complex and domain architecture of the PIK3CA-encoded p110α catalytic subunit. (**A**) Linear domain organization of the p110α catalytic subunit (PIK3CA). The protein comprises five highly conserved functional domains arranged from the N- to the C-terminus: the Adaptor-Binding Domain (ABD; aa 1–108), which mediates binding to the inter-Src homology 2 (iSH2; Inter-Src Homology 2) domain of the p85 regulatory subunit and contributes to stabilization of p110α; the RAS-Binding Domain (RBD; aa 108–186), responsible for interaction with guanosine triphosphate-bound RAS (RAS-GTP) and integration of RAS-dependent signaling; the C2 domain (Conserved Region 2; aa 186–291), which promotes phospholipid binding and membrane association; the Helical domain (aa 291–487), a key structural element involved in conformational regulation and a frequent site of activating mutations (e.g., E542K and E545K); and the Kinase domain (aa 487–1068), which contains the catalytic core responsible for phosphorylating phosphatidylinositol-4,5-bisphosphate (PIP2) into phosphatidylinositol-3,4,5-trisphosphate (PIP3) and harbors the common activating hotspot mutation H1047R. (**B**) The figure depicts the secondary structure-based organization of the p85α regulatory and p110α catalytic subunits in the autoinhibited state. Three major regulatory interfaces are highlighted: (1) the high-affinity interaction between the p85α iSH2 domain and the p110α adaptor-binding domain (ABD), which anchors and stabilizes the heterodimer; (2) additional contacts between iSH2 and the p110α C2 domain, contributing to catalytic regulation and membrane recruitment; and (3) the principal autoinhibitory interface between the p85α nSH2 domain and the p110α helical domain. The latter involves the helical-domain loop containing the oncogenic hotspot residues E542 and E545 and is complemented by contacts with the C2 and kinase domains. The enlarged panels illustrate the structural features of these three distinct interfaces.

PI3Ks are a family of lipid kinases that phosphorylate the 3′-hydroxyl group of the inositol ring of phosphoinositides, producing second messengers essential for signal transduction [7]. Based on their structure, substrate specificity, and regulatory mechanisms, PI3Ks are divided into three classes (I, II, and III), with Class I PI3Ks being the most extensively studied due to their central role in cancer biology [7,8]. Class I PI3Ks are heterodimeric complexes composed of a catalytic subunit (p110) and a regulatory subunit (p85) (Figure 1) [7,9].

The *PIK3CA* gene, located on chromosome 3q26.32, encodes the p110α catalytic subunit of class IA PI3Ks. This isoform is ubiquitously expressed and preferentially activated downstream of receptor tyrosine kinases (RTKs), such as epidermal growth factor receptor (EGFR), human epidermal growth factor receptor 2 (HER2), insulin-like growth factor 1 receptor (IGF1R), and fibroblast growth factor receptor (FGFR), as well as G protein-coupled receptors (GPCRs) through interactions with the p85 regulatory subunit [encoded by the phosphoinositide-3-kinase regulatory subunit 1 (*PIK3R1*), phosphoinositide-3-kinase regulatory subunit 2 (*PIK3R2*), or phosphoinositide-3-kinase regulatory subunit 3 (*PIK3R3*) genes] [7,9].The p110α protein is composed of five functional domains [7,9,10]:

1. Adaptor-binding domain (ABD): Located at the N-terminus, this domain interacts with the p85 regulatory subunit and is crucial for stabilizing the catalytic subunit in the cytoplasm.
2. Ras-binding domain (RBD): Mediates interaction with guanosine triphosphate-bound RAS (RAS-GTP), linking PI3K activity to RAS signaling.
3. C2 domain: Involved in membrane binding and lipid interaction, anchoring the protein to the inner leaflet of the plasma membrane.
4. Helical domain: Participates in conformational regulation and is frequently affected by oncogenic mutations (e.g., E542K, E545K).
5. Kinase domain: The C-terminal catalytic core responsible for the phosphorylation of phosphatidylinositol-4,5-bisphosphate (PIP2) into phosphatidylinositol-3,4,5-trisphosphate (PIP3). The H1047R mutation in this domain is one of the most common and functionally activating alterations in cancer.

The regulatory subunit p85 also contains multiple domains, including SH2 (Src homology 2) domains that recognize phosphotyrosine motifs on activated receptors or scaffolding proteins, and an inter-SH2 domain (iSH2) that binds tightly to the ABD of p110α. In its basal state, p85 stabilizes and inhibits p110α; upon receptor activation, p85 binds to the tyrosine-phosphorylated receptor docking sites, which releases its inhibitory grip, enabling catalytic activation of PI3K [7,9]. Importantly, structural studies using X-ray crystallography and cryo-EM have revealed that oncogenic mutations in the helical or kinase domains (see legend to Figure 1 for details) disrupt either the inhibitory interface between p110α and p85, or enhance plasma membrane association and lipid kinase activity, respectively, both leading to PI3K activation independent of upstream signals. These mutations can also promote membrane association or enhance substrate affinity, further contributing to hyperactivation of downstream effectors such as AKT and mTOR [9,10].

Overall, the modular and tightly regulated architecture of the PI3K complex allows for precise control of signaling in normal physiology. However, structural perturbations introduced by oncogenic mutations in *PIK3CA* result in sustained activation of growth-promoting and anti-apoptotic pathways, establishing p110α as a critical node in cancer pathogenesis and a rational target for therapeutic intervention.

## 3. Biology and Function of PIK3CA

**Figure 2:**
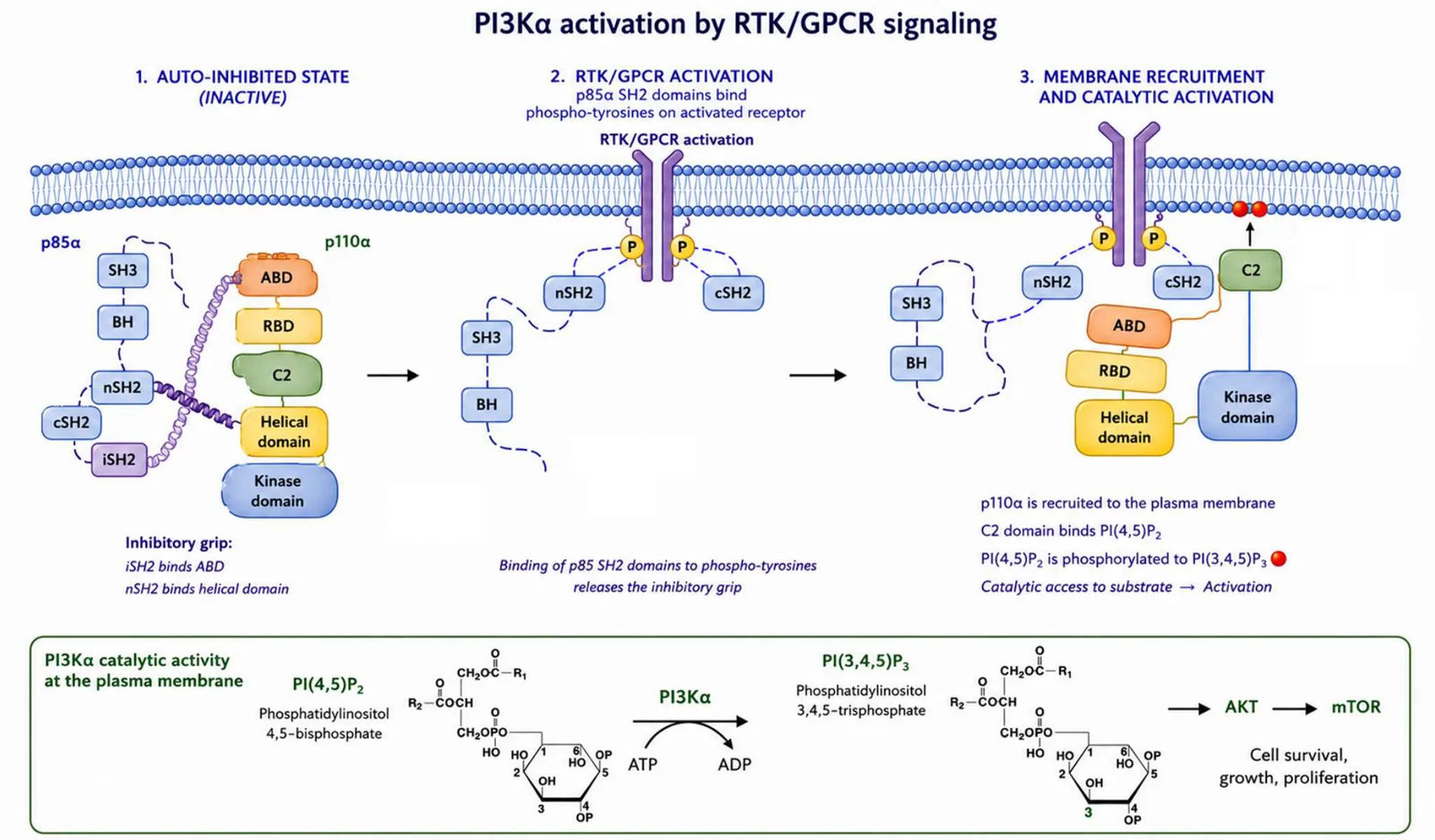
Canonical mechanism of activation of the Class IA phosphoinositide 3-kinase (PI3K)/protein kinase B (AKT)/mechanistic target of rapamycin (mTOR) signaling pathway. Following stimulation of receptor tyrosine kinases (RTKs) or G protein-coupled receptors (GPCRs), intracellular phosphotyrosine residues generated on activated receptors or adaptor proteins are recognized by the Src homology 2 (SH2) domains of the p85 regulatory subunit. This interaction relieves the inhibitory constraint exerted by p85 on the phosphatidylinositol-4,5-bisphosphate 3-kinase catalytic subunit alpha (PIK3CA)-encoded p110α catalytic subunit, promoting its recruitment to the inner leaflet of the plasma membrane and subsequent catalytic activation. Activated p110α phosphorylates phosphatidylinositol-4,5-bisphosphate (PIP2) to generate phosphatidylinositol-3,4,5-trisphosphate (PIP3), a key lipid second messenger that recruits proteins containing a pleckstrin homology (PH) domain, including AKT, to the plasma membrane. Activated AKT initiates multiple downstream signaling cascades, most notably the mTOR pathway, thereby promoting cell survival, growth, proliferation, and metabolic reprogramming. In tumors harboring activating PIK3CA mutations, this signaling cascade becomes constitutively active, resulting in persistent downstream signaling independently of upstream receptor stimulation.

The biological functions of p110α are primarily mediated through activation of the PI3K/AKT/mTOR signaling axis, a key pathway frequently dysregulated in human cancers [7,11,12]. The canonical mechanism of p110α activation and downstream signaling is summarized in Figure 2.

The production of PIP3 at the membrane serves as a docking site for pleckstrin homology (PH) domain-containing proteins, notably AKT and PDK1 (3-phosphoinositide-dependent protein kinase-1). Activated AKT phosphorylates a wide array of substrates involved in cell cycle progression [e.g., cyclin D1, cyclin-dependent kinase inhibitor 1A (p21^CIP1/WAF1), and cyclin-dependent kinase inhibitor 1B (p27^KIP1)], inhibition of apoptosis [e.g., BCL2-associated agonist of cell death (BAD), caspase-9, and Forkhead box O (FOXO) transcription factors], and metabolic reprogramming, including activation of mechanistic target of rapamycin complex 1 (mTORC1) through phosphorylation of tuberous sclerosis complex 2 (TSC2). This signaling axis is crucial for integrating environmental cues such as growth factors, nutrient availability, and stress signals, underscoring the central role of PI3K in maintaining cellular homeostasis [11,12,13].

Emerging evidence suggests that p110α also participates in non-canonical, kinase-independent functions. These include scaffolding interactions that facilitate complex formation at the membrane and regulation of cytoskeletal dynamics and vesicular trafficking [8].

PIK3CA is expressed in virtually all tissues but is particularly abundant in metabolically active organs such as the liver, muscle, and adipose tissue. In developmental biology, mouse models with conditional knockout of *PIK3CA* demonstrate embryonic lethality, highlighting its essential role in cell viability and organogenesis [14]. In adult physiology, p110α regulates glucose uptake and insulin signaling, angiogenesis, and tissue regeneration [7].

In the immune system, PI3K signaling modulates the development and activation of various leukocyte populations. For instance, in T cells, PI3K activation promotes survival, differentiation, and cytokine production, while in B cells, it regulates receptor-mediated signaling crucial for antibody class switching and maturation. In endothelial cells, p110α regulates vascular permeability and neovascularization, partly through AKT-mediated phosphorylation of endothelial nitric oxide synthase (eNOS) and modulation of vascular endothelial growth factor (VEGF) signaling [15].

The PI3K pathway is embedded in a complex network of feedback loops and crosstalk with other signaling cascades. One prominent example is the negative feedback from mTORC1 to insulin receptor substrate (IRS) proteins, which modulates upstream PI3K activation [11,12,13]. Additionally, PTEN (phosphatase and tensin homolog) antagonizes PI3K function by dephosphorylating PIP3 back to PIP2, thereby acting as a critical tumor suppressor and maintaining PI3K pathway homeostasis [7,16].

PI3K also exhibits extensive crosstalk with the RAS/mitogen-activated protein kinase (MAPK) signaling pathway through direct interaction of RAS-GTP with the RBD of p110α, as well as through downstream signaling intermediates that regulate extracellular signal-regulated kinase (ERK) activity. This functional redundancy and compensation explain, in part, why dual inhibition of both pathways is often necessary for effective anti-cancer therapy in PI3K-driven tumors [9,12].

## 4. Cancers with *PIK3CA* Alterations and Their Oncogenic Role

Mutations in *PIK3CA* are among the most recurrent somatic alterations in human malignancies, driving oncogenesis through multifaceted mechanisms that affect nearly every hallmark of cancer. Their high prevalence is a consequence of both the functional impact of PI3K pathway activation and the adaptive advantages conferred to evolving tumor clones [17]. The oncogenic activation of the PI3K/AKT/mTOR pathway via *PIK3CA* mutation or amplification provides a selective advantage during tumor initiation and progression by promoting cell proliferation, metabolic rewiring, and anti-apoptotic signaling, which leads to resistance to stress and therapy. Here, we provide an overview of the cancer types most frequently harboring *PIK3CA* alterations, the mechanistic implications of these alterations for tumor biology, and the reasons for their prevalence and selective advantage in the cancer context.

### 4.1. Spectrum of PIK3CA Alterations in Cancer

*PIK3CA* alterations in cancer are diverse and extend beyond classical hotspot point mutations, encompassing a broad range of genomic events that collectively contribute to aberrant PI3K pathway activation. These alterations can be classified into several main categories: missense mutations at hotspot residues, copy number variations (CNVs), gene amplifications, rare non-hotspot mutations, and structural variants [18]. Understanding the spectrum of these genomic changes is critical for deciphering the mechanisms of oncogenic PI3K signaling and for optimizing targeted therapeutic strategies.

The majority of oncogenic *PIK3CA* mutations cluster within two key functional domains of the p110α catalytic subunit: the helical domain (exon 9) and the kinase domain (exon 20). The three most frequent hotspots include: E542K and E545K in the helical domain and H1047R in the kinase domain. These mutations increase the level of lipid kinase activity by disrupting the interaction with the regulatory subunit p85α or by directly enhancing catalytic function, resulting in persistent downstream activation of AKT and mTOR signaling. Hotspot mutations have been extensively validated in preclinical models and are the primary targets of PI3Kα-specific inhibitors currently in clinical use [6].

Beyond hotspots, *PIK3CA* harbors a spectrum of less frequent mutations scattered throughout the gene, including missense mutations in exons 1, 4, 7, and 13. While individually rare, collectively these variants may contribute to oncogenicity through diverse mechanisms such as altered protein stability, subcellular localization, or interaction with partner proteins. Functional characterization of these mutations remains limited but is increasingly recognized as necessary for comprehensive genomic interpretation [19]. From a clinical sequencing perspective, however, the detection of a rare *PIK3CA* variant should not be equated automatically with oncogenic activation or therapeutic actionability. Current somatic-variant interpretation relies on an integrated, evidence-based framework that considers population frequency, recurrence in cancer datasets, localization within established functional domains or mutational hotspots, computational and structural predictions, experimental functional data, and clinical evidence of drug response. Consensus recommendations from ClinGen, the Cancer Genomics Consortium, and the Variant Interpretation for Cancer Consortium classify somatic variants according to the strength of evidence supporting oncogenicity, while knowledge bases and interpretation platforms such as the Cancer Genome Interpreter can complement manual curation by integrating driver prediction and available therapeutic evidence [20,21]. Importantly, in silico annotation alone is insufficient for rare variants of uncertain significance (VUS), and functional validation remains particularly valuable when clinical evidence is sparse. Assays assessing PI3K/AKT pathway activation, cellular transformation, growth-factor-independent signaling, drug sensitivity, and increasingly high-throughput mutational approaches can distinguish activating from neutral variants. This distinction is clinically relevant for *PIK3CA*: in HNSCC, systematic functional characterization of 32 noncanonical mutations showed that 69% were activating, and a patient harboring the noncanonical Q75E variant experienced a durable response to alpelisib, illustrating that potentially actionable alterations may extend beyond classical hotspots [22,23]. Nevertheless, functional activation does not by itself establish clinical predictiveness across tumor types; lineage, allelic configuration, clonality, co-mutations, and the specific PI3K inhibitor remain important determinants of therapeutic response. Accordingly, rare *PIK3CA* variants identified by next-generation sequencing should be interpreted through convergent genomic, computational, structural, functional, and clinical evidence rather than by mutation position alone.

*PIK3CA* gene amplification is observed in a subset of tumors, often correlating with increased mRNA and protein expression levels. Amplifications tend to be more frequent in breast, ovarian, and head and neck cancers and can coexist with point mutations. While amplification alone may modestly activate the PI3K pathway, it may also potentiate the oncogenic effects of co-occurring mutations [24] and has been associated with poor prognosis in certain cancers and may modulate sensitivity to PI3K pathway inhibitors [25,26,27].

Though rare, *PIK3CA* structural rearrangements such as gene fusions have been described, especially in pediatric tumors and sarcomas. These fusions often result in aberrant PI3K activity via constitutive activation or altered regulation. Examples include fusions with regulatory partners that promote constitutive membrane localization or bypass normal feedback inhibition. The clinical relevance of *PIK3CA* fusions is an emerging area of research, with implications for novel targeted approaches [28].

The oncogenic potential of *PIK3CA* alterations is frequently modulated by co-mutations in other genes within the PI3K/AKT/mTOR axis or parallel signaling pathways such as RAS/RAF/MEK. Co-occurrence with *PTEN* loss, *KRAS* mutations, or *TP53* alterations is common and can influence both tumor aggressiveness and therapeutic responses. For instance, *PIK3CA* mutations coexisting with *KRAS* mutations are associated with more aggressive phenotypes and resistance to EGFR-targeted therapies, as observed in CRC [6,19,29].

### 4.2. Prevalence and Distribution of PIK3CA Mutations Across Cancer Types

Large-scale genomic resources and clinical sequencing cohorts, including The Cancer Genome Atlas (TCGA), Memorial Sloan Kettering–Integrated Mutation Profiling of Actionable Cancer Targets (MSK-IMPACT), and the Catalogue Of Somatic Mutations In Cancer (COSMIC), consistently identify *PIK3CA* as one of the most frequently altered oncogenes across solid tumors [30,31,32]. However, a single pan-cancer prevalence figure is potentially misleading because estimates depend on cohort composition, tumor lineage and subtype, disease stage, specimen type, sequencing platform, assay coverage, and whether copy-number changes are included. For example, a large pan-cancer analysis of 352,392 tumor samples reported *PIK3CA* mutations in 12.1% of specimens, illustrating the dataset dependence of global estimates [19]. Accordingly, the frequencies reported in Table 1 are presented as approximate descriptive ranges rather than directly comparable point estimates.

#### 4.2.1. Breast Cancer

*PIK3CA* mutations are highly prevalent in breast cancer, particularly within hormone receptor-positive (HR+)/HER2-negative subtypes, where mutation frequencies reach 30–40% [33,34]. They are also detected, albeit to a lesser extent, in HER2-positive and triple-negative breast cancers (TNBC) [35,36]. In HR+ tumors, the frequent co-activation of the estrogen receptor (ER) and PI3K/AKT/mTOR pathways underlies both ligand-independent proliferation and adaptive resistance to endocrine therapies [37]. The prognostic significance of *PIK3CA* mutation in breast cancer is, however, context-dependent rather than uniformly favorable. In early-stage disease, large pooled analyses have associated *PIK3CA* mutations with more favorable clinicopathologic features and improved invasive disease-free survival, although the independent effect is attenuated after adjustment for conventional prognostic variables [38,39]. Conversely, in advanced HR+/HER2-negative breast cancer not treated with PI3K-targeted agents, a meta-analysis of clinical-trial cohorts associated *PIK3CA* mutations with shorter PFS and OS [40]. Moreover, mutation-domain analyses suggest that helical- and kinase-domain alterations may not carry identical prognostic information [41]. Thus, disease stage, molecular subtype, treatment exposure, and mutation domain should be considered when interpreting PIK3CA as a prognostic biomarker. PIK3CA-mutant tumors may also show reduced sensitivity to selected systemic treatments, emphasizing the distinction between prognostic and predictive effects [42]. Clinical trials with PIK3CA-specific inhibitors, such as alpelisib, have shown improved progression-free survival (PFS) specifically in patients with *PIK3CA*-mutant HR+ disease, reinforcing the biomarker’s utility in precision oncology [43].

#### 4.2.2. Endometrial Carcinoma

Among all cancers, endometrial carcinoma exhibits one of the highest frequencies of *PIK3CA* mutations, with estimates approaching 50%, particularly in the endometrioid subtype [44,45]. These mutations commonly co-occur with *PTEN* loss or inactivating mutations, resulting in synergistic hyperactivation of the PI3K cascade [46]. Importantly, in this setting, *PIK3CA* mutations correlate with PI3K-dependence and may identify tumors likely to respond to PI3K or dual PI3K/mTOR inhibitors [45,46]. Given their frequent occurrence in early-stage disease, *PIK3CA* alterations may also serve as molecular markers of precancerous transformation, opening avenues for preventive strategies [47]. From a prognostic perspective, however, the evidence is not definitive. A systematic review and meta-analysis found only a non-significant trend toward impaired survival for tumors harboring exon 9 and/or exon 20 *PIK3CA* mutations (RR 1.28, 95% CI 0.84–1.94), with substantial context dependence across histologic type and grade [48]. Individual studies have further suggested that adverse associations may be concentrated in specific molecular or histologic subsets, including grade 3 endometrioid carcinoma and selected exon 9 alterations, rather than representing a universal effect of *PIK3CA* mutation [49,50].

#### 4.2.3. Cervical Cancer

In cervical squamous cell carcinoma (CSCC) and cervical adenocarcinoma (CAC), *PIK3CA* mutations are detected in approximately 25–40% of cases [51]. These alterations frequently arise early during human papillomavirus (HPV)-driven carcinogenesis and can already be identified in preinvasive lesions such as cervical intraepithelial neoplasia (CIN). Their early occurrence suggests that PIK3CA activation contributes to viral-mediated tumorigenesis, likely acting downstream of the E6 and E7 viral oncoproteins [52,53]. Functionally, these alterations promote sustained proliferative signaling and facilitate escape from growth-suppressive mechanisms. Clinically, a subset of these tumors may exhibit varying degrees of dependence on PI3K pathway signaling, providing a biological basis for the investigation of PI3K-targeted therapeutic strategies.

#### 4.2.4. Colorectal Cancer (CRC)

In colorectal adenocarcinomas, *PIK3CA* mutations are observed in 15–20% of cases and show preferential localization in proximal (right-sided) tumors and those with mucinous histology [29,54,55]. These mutations frequently coexist with *KRAS* or *BRAF* (B-Raf proto-oncogene, serine/threonine kinase) mutations and have been associated with resistance to anti-EGFR monoclonal antibodies (e.g., cetuximab and panitumumab), especially in the setting of dual PI3K and RAS/RAF pathway activation. The presence of *PIK3CA* mutations alone does not universally predict resistance, but co-occurrence with *KRAS* mutations has emerged as a significant biomarker of therapeutic failure with anti-EGFR strategies [56,57]. Therefore, assessing the combinatorial mutational landscape is crucial for accurate treatment planning. The prognostic effect is more nuanced than a simple adverse association. In metastatic CRC, *PIK3CA*-mutant tumors have been associated with shorter overall survival (OS) in a contemporary cohort [58], but earlier large series showed that the effect may depend on mutation pattern: concomitant exon 9 and exon 20 mutations were associated with markedly worse cancer-specific and OS, whereas a mutation in either exon alone was not [59]. In stage III colon cancer, exon 20 kinase-domain mutations have likewise been linked to poorer disease-free and cancer-specific survival, an association not observed in stage I–II disease [60]. These data indicate that stage, exon/domain, co-mutations, (microsatellite instability) MSI status, and tumor sidedness can materially modify the prognostic meaning of *PIK3CA* alterations.

#### 4.2.5. Head and Neck Squamous Cell Carcinoma (HNSCC)

In HNSCC, mutation frequencies range from 20–25%, with a predilection for HPV-positive tumors, where *PIK3CA* mutations act in concert with viral oncogene expression [61,62]. The biological relevance of PI3K pathway activation and its frequent involvement in HPV-positive HNSCC strongly support PI3K inhibition as a promising therapeutic strategy deserving further prospective validation in biomarker-driven clinical trials [22,63]. Importantly, mutation status should not be interpreted as an established adverse prognostic marker in HNSCC. A systematic review/meta-analysis reported heterogeneous results and an overall trend toward improved prognosis in *PIK3CA*-mutant tumors; in the accompanying cohort, mutation status was not significantly associated with OS, whereas HPV status remained the dominant prognostic factor, with evidence of an interaction between HPV and PIK3CA for PFS [62]. Accordingly, HPV status and anatomic/molecular subgroup are major potential confounders when evaluating the independent prognostic contribution of *PIK3CA*.

Lung Cancer. In non-small cell lung cancer (NSCLC), particularly adenocarcinomas, *PIK3CA* mutations occur in approximately 2–4% of cases [64]. These are often subclonal events, and their biological role is less well defined. Nonetheless, *PIK3CA* mutations may contribute to acquired resistance to EGFR tyrosine kinase inhibitors (TKIs) or other targeted agents, particularly in post-treatment biopsies, where selection pressure may enrich for PI3K-activated subpopulations [65]. The prognostic literature is likewise heterogeneous. In surgically treated or systemically treated NSCLC, *PIK3CA* mutations have not consistently shown an independent negative impact on survival [66], whereas in EGFR-mutant disease treated with EGFR-TKIs, concurrent *PIK3CA* mutations have been associated with shorter PFS and OS [67]. Conversely, a cohort of early-stage squamous cell lung cancers reported longer OS and PFS in *PIK3CA*-mutant cases [68]. These apparently discordant findings reinforce the need to distinguish histology, co-driver status, disease stage, and treatment context.

#### 4.2.6. Ovarian Cancer

*PIK3CA* mutations are relatively enriched in clear cell and endometrioid ovarian carcinomas, with mutation rates of 20–30%, while they are rare in high-grade serous ovarian carcinomas [69,70]. These mutations likely participate in early tumorigenesis, and preclinical studies suggest that clear cell ovarian tumors may exhibit functional PI3K pathway dependency, making them attractive candidates for pathway-specific inhibition [70]. Prognostic data in ovarian clear cell carcinoma are also inconsistent. One clinicopathologic series associated *PIK3CA* mutation with favorable OS [70], whereas a later molecularly stratified cohort found *PIK3CA* mutation, particularly when considered together with *ARID1A* status, to be associated with disease progression and impaired stage-adjusted PFS/OS [71]. Therefore, *PIK3CA* alone cannot currently be regarded as a robust directionally consistent prognostic marker in this histotype.

#### 4.2.7. Bladder and Urothelial Cancers

Approximately 15–20% of bladder tumors harbor *PIK3CA* mutations, frequently co-occurring with mutations in the telomerase reverse transcriptase (TERT) promoter and the fibroblast growth factor receptor 3 (FGFR3) gene [72,73]. Their frequent co-occurrence in non-muscle-invasive bladder cancer suggests a role in early bladder carcinogenesis, although their prognostic impact remains unclear [73]. Nonetheless, PI3K/AKT inhibitors are being explored in this setting.

#### 4.2.8. Gliomas

Although relatively rare, *PIK3CA* mutations can be detected in pediatric gliomas and *IDH* (isocitrate dehydrogenase)-mutant low-grade gliomas, where they may contribute to treatment resistance, particularly to radiotherapy and alkylating agents [74]. The functional relevance in gliomagenesis is still under investigation.

#### 4.2.9. Other Solid Tumors

Lower-frequency mutations in *PIK3CA* have also been described in gastric, esophageal, prostate, pancreatic, and hepatocellular carcinomas, though typically within genomically defined subsets. For instance, *PIK3CA* mutations in gastric cancer may enrich for Epstein–Barr virus–positive tumors [75], while in prostate cancer they often emerge as subclonal escape mechanisms during androgen receptor-targeted therapy [76]. Interestingly, in gastric cancer, a meta-analysis found no significant association between *PIK3CA* gene mutation and OS, despite an adverse prognostic association for high PIK3CA protein expression [77].

Overall, the available evidence argues against assigning a single pan-cancer prognostic direction to *PIK3CA* mutations. Their clinical meaning depends on tumor lineage, disease stage, hotspot/domain, clonality, co-alterations, and treatment exposure; consequently, prognostic associations should be separated conceptually from predictive sensitivity or resistance to PI3K-directed and other systemic therapies.

### 4.3. Oncogenic Role and Functional Consequences of PIK3CA Alterations

The oncogenic behavior of *PIK3CA* mutations is fundamentally rooted in their capacity to enforce a persistent activation of the PI3K signaling cascade, independent of extracellular stimuli. Unlike the tightly regulated, ligand-dependent activation observed in physiological contexts, oncogenic mutations in *PIK3CA*—particularly those affecting the helical and kinase domains of p110α— promote a global increase in enzymatic activity that leads to continuous production of PIP3 [9,12]. This, in turn, promotes chronic activation of downstream effectors such as AKT and mTOR, thereby enabling a broad range of cellular programs that support neoplastic transformation and progression [12].

One of the most immediate consequences of this aberrant activation is a marked enhancement of cell survival. Activated AKT phosphorylates and inactivates several pro-apoptotic proteins, including members of the BCL-2 family such as BAD, and critical components of the intrinsic apoptotic pathway like caspase-9 [78]. Additionally, AKT activity leads to the cytoplasmic retention of FOXO transcription factors, which under normal conditions translocate to the nucleus to promote the expression of genes involved in cell cycle arrest and apoptosis [79,80]. The net result is an environment in which cells are shielded from death-inducing stimuli and equipped to withstand various forms of cellular stress.

This survival advantage is paralleled by a significant deregulation of proliferative control. The sustained activation of AKT enhances the expression of cyclin D1, a key driver of G1/S transition, while simultaneously suppressing the expression and function of CDK inhibitors such as p21^CIP1 and p27^KIP1 [81]. In addition, mTORC1, a critical regulator of anabolic processes and protein synthesis, is activated downstream of AKT, further fueling the biosynthetic machinery required for continuous cell division [82]. Collectively, these alterations disrupt the normal checks and balances of cell cycle control, allowing for unchecked cellular proliferation. However, beyond survival and proliferation, *PIK3CA* mutations also instigate profound changes in cellular metabolism, consistent with the metabolic reprogramming that characterizes malignant cells. Mutant *PIK3CA* signaling upregulates glucose transporter expression [notably GLUT1 (glucose transporter 1)], increases glycolytic flux, and enhances lipid biosynthesis [83]. These metabolic adaptations, often referred to as the Warburg effect, allow cancer cells to maintain high proliferative rates even under hypoxic or nutrient-limited conditions by promoting biomass accumulation and maintaining redox balance [84]. A further hallmark of PIK3CA-driven oncogenesis is the promotion of angiogenesis and invasive potential. Sustained PI3K signaling enhances the transcription and secretion of VEGF, a potent pro-angiogenic cytokine that facilitates neovascularization and increases vascular permeability [85,86]. In parallel, the expression of matrix-degrading enzymes such as matrix metalloproteinases (MMPs) is upregulated, thereby disrupting the extracellular matrix and promoting local invasion and metastatic dissemination [87].

### 4.4. PIK3CA Mutations, Tumor Microenvironment, and Anti-Tumor Immunity

Accumulating evidence indicates that the oncogenic consequences of *PIK3CA* mutations extend well beyond cancer cell-intrinsic proliferation and survival, profoundly influencing the tumor immune microenvironment (TME). Increased activation of the PI3K/AKT/mTOR axis reshapes the cytokine and chemokine landscape, generating an immunosuppressive milieu that impairs effective anti-tumor immune surveillance [88,89]. In particular, aberrant PI3K signaling reduces the production of chemokines involved in the recruitment of cytotoxic lymphocytes while increasing the expression of soluble mediators that favor the accumulation and activation of immunosuppressive myeloid populations. Consequently, tumors harboring activating *PIK3CA* mutations exhibit reduced infiltration and impaired activation of CD8+ T lymphocytes together with increased recruitment of myeloid-derived suppressor cells (MDSCs), regulatory T cells (Tregs), and alternatively activated tumor-associated macrophages (TAMs), all of which cooperate to suppress adaptive immune responses and promote tumor progression [88,89]. Mechanistically, this remodeling of the cytokine and chemokine landscape can be traced to transcriptional nodes downstream of, or functionally coupled to, PI3K–AKT–mTOR signaling. AKT can potentiate nuclear factor-κB (NF-κB) transcriptional activity through phosphorylation-dependent mechanisms involving the IκB kinase complex and NF-κB subunits, thereby linking PI3K activation to the transcription of inflammatory and immune-regulatory genes [90]. NF-κB also engages in extensive crosstalk with signal transducer and activator of transcription 3 (STAT3): NF-κB-dependent cytokines, particularly interleukin-6 (IL-6), can activate STAT3 in tumor and immune cells, while cooperative NF-κB/STAT3 activity sustains feed-forward transcriptional programs that reinforce tumor-promoting inflammation and immune suppression [91]. In parallel, PI3K–AKT–mTOR signaling can enhance hypoxia-inducible factor 1α (HIF-1α) activity through oxygen-independent mechanisms, providing an additional transcriptional route linking oncogenic signaling to glycolytic, angiogenic, and microenvironmental adaptation [92]. Thus, activating *PIK3CA* alterations should not be viewed as merely changing cytokine abundance downstream; rather, they can rewire cytokine and chemokine expression through intersecting NF-κB-, STAT3-, and HIF-1α-centered transcriptional programs, the relative contribution of which is likely to remain tumor- and context-dependent.

In parallel, activation of PI3K signaling directly contributes to immune escape through the regulation of immune checkpoint pathways. Activation of the PI3K/AKT/mTOR cascade has been consistently associated with increased expression of programmed death-ligand 1 (PD-L1) on tumor cells and, in some settings, on infiltrating myeloid cells, thereby promoting T-cell exhaustion through the programmed cell death protein 1 (PD-1)/PD-L1 immune checkpoint axis and impairing endogenous antitumor immunity [93,94,95]. Importantly, growing preclinical evidence indicates that pharmacological inhibition of PI3K not only suppresses oncogenic signaling but also reprograms the TME by reducing immunosuppressive myeloid cells, restoring CD8+ T-cell infiltration and effector function, and enhancing the activity of immune checkpoint blockade. In cervical cancer models, for example, selective PI3K inhibition significantly potentiated the therapeutic efficacy of anti-PD-1 antibodies, providing a biological rationale for combining PI3K inhibitors with immunotherapy [94]. These observations should, however, be interpreted primarily as preclinical evidence of pharmacological synergy rather than as evidence of established clinical benefit. Although early-phase clinical investigation of PI3K inhibition combined with immune checkpoint blockade has begun, the available human data remain limited. For example, a phase I/II study of the PI3Kβ inhibitor GSK2636771 plus pembrolizumab in *PTEN*-loss metastatic castration-resistant prostate cancer was designed primarily to establish safety and a recommended phase II dose, with antitumor activity assessed in a small, molecularly selected cohort [96]. Thus, such studies are hypothesis-generating and cannot yet establish that PI3K inhibition improves the efficacy of immune checkpoint blockade in routine clinical practice.

Recent experimental evidence has further demonstrated that *PIK3CA* mutations orchestrate immune remodeling through metabolic reprogramming of the TME. Hyperactivation of PI3Kα induces a Warburg-like metabolic phenotype characterized by increased aerobic glycolysis, upregulation of glycolytic regulators, including HIF-1α, MYC proto-oncogene, and excessive lactate production. Beyond supporting tumor bioenergetics, lactate acts as an immunomodulatory metabolite that promotes macrophage recruitment and polarization toward an M2/TAM-like phenotype, facilitates the accumulation of lipid-associated triggering receptor expressed on myeloid cells 2-positive (TREM2+) and lymphatic vessel endothelial hyaluronan receptor 1-positive (Lyve1+) macrophages, promotes the expansion of MDSCs, and reduces the infiltration of both cluster of differentiation 8-positive (CD8+) T lymphocytes and natural killer (NK) cells. These changes are accompanied by reduced systemic and local levels of pro-inflammatory cytokines, including interferon-γ (IFN-γ), tumor necrosis factor-α (TNF-α), and interleukin-6 (IL-6), together with extracellular matrix remodeling, collagen deposition, and fibrosis, ultimately establishing an immune-tolerant niche that closely resembles the tumor microenvironment of established malignancies [97]. Importantly, these mechanistic observations derive from experimental systems and demonstrate biological plausibility, but they do not by themselves establish that PIK3CA-driven immune remodeling is therapeutically reversible in patients or that such reversal translates into improved response or survival with immune checkpoint inhibitors.

**Figure 3:**
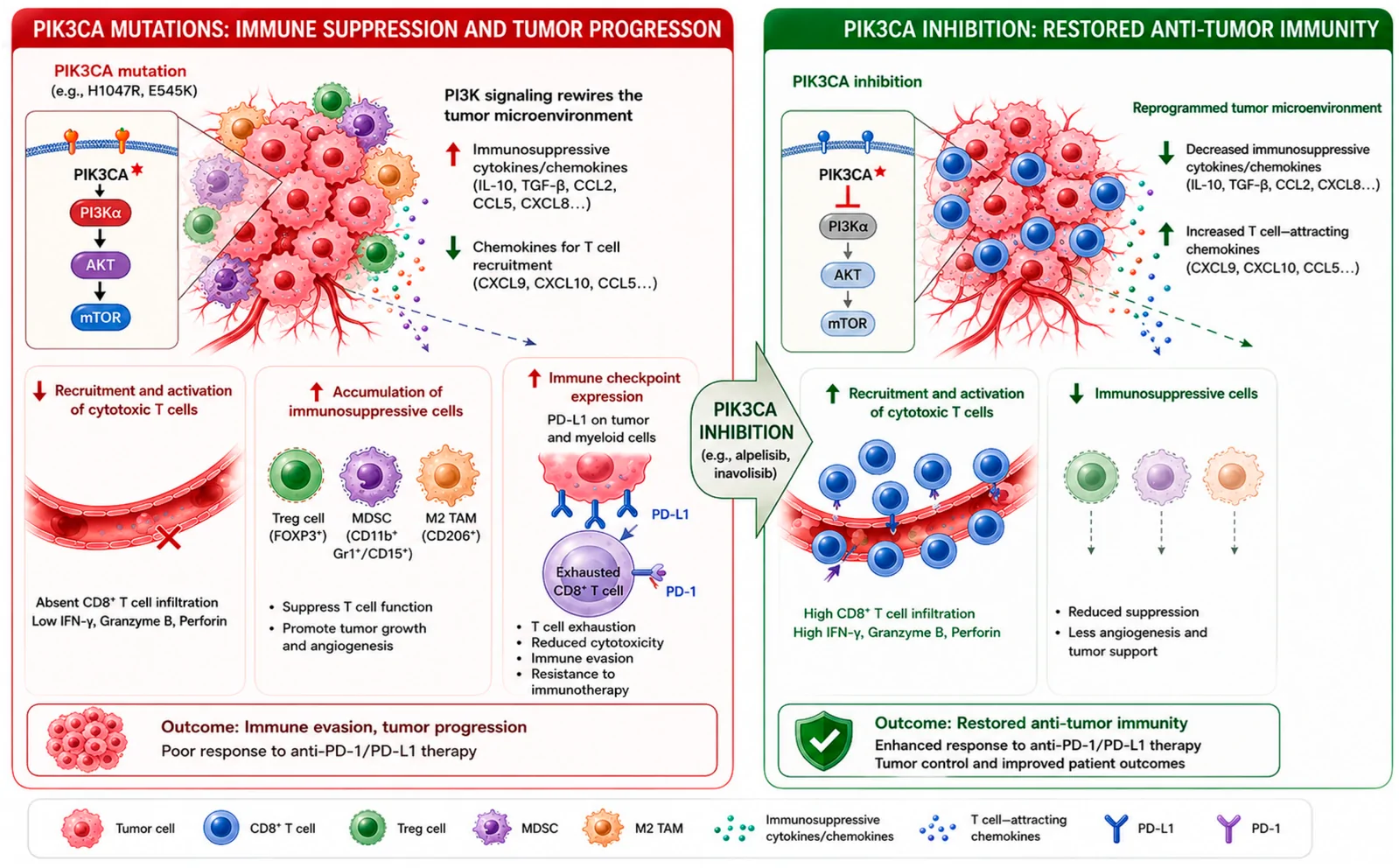
*PIK3CA* mutations and potential modulation of the antitumor tumor microenvironment. The figure provides a schematic representation of the proposed and predominantly preclinically supported relationships between activating *PIK3CA* mutations, tumor immune escape, and the potential immune-restorative effects of PI3Kα inhibition. The depicted mechanisms should not be interpreted as uniformly established across tumor types, as their magnitude and biological relevance may vary according to tumor lineage, molecular context, experimental model, and treatment setting. (**Left**) panel: *PIK3CA* mutations—potential mechanisms of immune suppression and tumor progression. Activating mutations, such as H1047R or E545K, result in sustained activation of the phosphoinositide 3-kinase alpha (PI3Kα)–protein kinase B (AKT)–mechanistic target of rapamycin (mTOR) signaling axis in tumor cells. Experimental studies suggest that, in selected biological contexts, this oncogenic signaling may contribute to remodeling of the tumor microenvironment through altered production of cytokines and chemokines, including reduced signals involved in the recruitment of cytotoxic cluster of differentiation 8-positive (CD8+) T lymphocytes. Preclinical models have consequently reported reduced CD8+ T-cell infiltration and/or activity, accompanied by decreased production of effector molecules, including interferon-γ (IFN-γ), granzyme B, and perforin. PI3K pathway activation has also been associated in experimental systems with the accumulation or functional polarization of immunosuppressive cell populations, including myeloid-derived suppressor cells (MDSCs), regulatory T cells (Tregs), and M2-like tumor-associated macrophages (TAMs), potentially contributing to impaired T-cell function, tumor growth, and angiogenesis. In addition, PI3K pathway activation has been linked, in a context-dependent manner, to increased expression of immune checkpoint molecules, including programmed death-ligand 1 (PD-L1) on tumor and/or myeloid cells, which may favor CD8+ T-cell dysfunction through programmed cell death protein 1 (PD-1) signaling. Collectively, these mechanisms provide a biological framework through which PIK3CA activation could contribute to immune evasion and tumor progression; however, their relative contribution in human tumors and their impact on clinical responsiveness to anti-PD-1/PD-L1 immune checkpoint blockade remain incompletely defined. (**Right**) panel: PI3Kα inhibition—potential restoration of antitumor immunity. Pharmacological inhibition of PI3Kα attenuates downstream AKT–mTOR signaling and, in several experimental models, has been shown to partially reverse selected immunosuppressive features of the tumor microenvironment. These effects may include reduced production of immunosuppressive cytokines and chemokines, increased expression of T-cell-attracting signals, and enhanced recruitment and activation of cytotoxic CD8+ T lymphocytes. Preclinical studies have also described restoration of T-cell effector functions, including increased production of IFN-γ, granzyme B, and perforin, together with reductions in the abundance and/or suppressive activity of MDSCs, Tregs, and M2-like TAMs. Such changes could theoretically reduce immune suppression and pro-angiogenic support and improve immune-mediated tumor control. Overall, these findings provide a biological and preclinical rationale for combining PI3Kα inhibition with immune checkpoint blockade.

Collectively, these findings support PIK3CA as an important regulator of both tumor cell biology and immune ecosystem remodeling (Figure 3). Through the coordinated regulation of metabolism, cytokine production, extracellular matrix organization, immune checkpoint expression, and immune cell composition, activating *PIK3CA* mutations generate a tumor-permissive microenvironment that favors immune evasion and therapeutic resistance. Conversely, preclinical models indicate that PI3K inhibition may reverse several of these alterations, transforming an immunologically “cold” tumor into a more inflamed and immune-responsive microenvironment and thereby providing a compelling biological rationale for integrating PI3K-targeted agents with immune checkpoint inhibitors in biomarker-selected patients. At present, however, the evidentiary hierarchy remains clearly unbalanced: the biological rationale is well supported and the preclinical evidence is substantial, but clinical validation remains preliminary. No definitive randomized evidence currently demonstrates that adding a PI3K inhibitor to PD-1/PD-L1 blockade improves clinically meaningful outcomes specifically because of *PIK3CA* mutation or PI3K-pathway activation. Moreover, pathway complexity, isoform-specific effects on tumor and immune cells, treatment-related toxicity, and the absence of a validated predictive biomarker may limit translation of preclinical synergy into clinical benefit. Accordingly, PI3K–immune checkpoint combinations should currently be regarded as an investigational strategy requiring prospective biomarker-driven validation rather than as an established therapeutic approach.

### 4.5. Why PIK3CA Mutations Are Frequent and Favored in Cancer

The remarkable prevalence of *PIK3CA* mutations across a wide spectrum of human malignancies reflects not only the centrality of the PI3K pathway in cellular physiology but also the selective advantages these alterations confer during oncogenesis. One of the key reasons for their frequency lies in the early clonal advantage they provide. In many tumor types, activating mutations in *PIK3CA* arise at initial stages of neoplastic transformation, particularly within proliferative epithelial compartments. These mutations promote increased cell survival, proliferative autonomy, and metabolic support, enabling the mutant clone to outcompete neighboring cells and progressively dominate the developing lesion. The persistence of such clones through subsequent stages of progression indicates their fitness in evolving tumor ecosystems [98].

Another aspect that favors the positive selection of *PIK3CA* mutations is the biological tolerance of the p110α protein to structural alterations. Unlike more rigid oncogenes such as *RAS* or *MYC*, where hyperactivation may lead to oncogene-induced senescence or apoptosis, p110α can accommodate activating mutations—such as E545K or H1047R—without immediately disrupting cellular homeostasis. This “modularity” enables mutant PI3K signaling to enhance growth without crossing thresholds that would otherwise trigger defensive cellular programs. As a result, *PIK3CA* mutations can act as “safe” drivers, pushing cells toward transformation while avoiding catastrophic signaling overload [99,100].

Moreover, the cooperativity between *PIK3CA* mutations and other oncogenic lesions further enhances their selection during tumorigenesis. These mutations frequently co-occur with alterations in *TP53*, *KRAS*, *BRAF*, or loss of tumor suppressors such as *PTEN*, particularly in cancers like colorectal, breast, and endometrial carcinomas [101]. Such combinations result in synergistic oncogenic signaling that supports not only tumor initiation but also progression and resistance to stress. The interaction with pathways such as RAS/MAPK or WNT [family of secreted signaling proteins, derived historically from the fusion of the Wingless (Wg) gene in Drosophila and the Int-1 gene in mice] contributes to a more robust and adaptive tumor phenotype.

In specific tissue contexts—particularly hormone-responsive organs such as the breast and endometrium—*PIK3CA* mutations exhibit a unique advantage. In these settings, PI3K signaling interacts intimately with estrogen or progesterone receptor activity, amplifying the effects of hormone-dependent proliferation. This functional crosstalk contributes to therapeutic resistance, especially in hormone receptor-positive breast cancers, where *PIK3CA*-mutant clones may persist or expand under endocrine treatment pressure [102,103].

Finally, there is growing evidence that in certain tumor types *PIK3CA* mutations may emerge later as subclonal events, particularly in response to therapeutic interventions. In *EGFR*-mutant lung cancers or HER2-positive breast cancers, *PIK3CA* mutations appear in resistant subpopulations following targeted therapy [104,105,106]. This adaptive selection reflects the capacity of PI3K pathway activation to bypass upstream inhibition and sustain survival signaling in the face of pharmacologic stress, contributing to acquired resistance and disease recurrence.

The frequent selection of *PIK3CA* mutations in cancer reflects a convergence of evolutionary factors: their early oncogenic potential, biological compatibility with sustained signaling, functional synergy with co-mutations, context-dependent amplification of growth signals, and adaptability under therapeutic pressure. These features establish *PIK3CA* not merely as a common mutational target, but as a versatile and highly exploitable node in the malignant signaling network.

### 4.6. Preclinical Models of PIK3CA-Driven Cancer

The oncogenic potential of *PIK3CA* has been extensively investigated using a variety of genetically engineered mouse models (GEMMs) and functional in vitro systems. These models have been instrumental not only in confirming the transforming capability of specific *PIK3CA* mutations, but also in elucidating their cooperative behavior with other oncogenic or tumor suppressor alterations within defined tissue compartments [107,108].

One of the most extensively investigated mutations, H1047R, located within the kinase domain of p110α, has been introduced into the mouse genome under the control of tissue-specific promoters using conditional Cre (Causes recombination) recombinase–loxP (Cre-loxP) systems. In the mammary epithelium, mouse models driven by the mouse mammary tumor virus (MMTV)-Cre or whey acidic protein (WAP)-Cre promoters have demonstrated that expression of the *PIK3CA* H1047R allele is sufficient to initiate neoplastic transformation, leading to the development of multifocal mammary tumors that closely recapitulate the histopathological features of human luminal breast cancer [109]. These tumors often display high AKT phosphorylation and are enriched in stem/progenitor-like populations, highlighting the impact of PI3K signaling on both differentiation and self-renewal.

Similarly, in the endometrial epithelium, expression of the PIK3CA H1047R allele under the control of the lactotransferrin (Ltf)-Cre system results in glandular hyperplasia and progression to endometrial carcinoma, providing direct evidence that PIK3CA activation alone is sufficient to initiate tumorigenesis in hormone-responsive tissues [110]. Importantly, these models also recapitulate hormonal crosstalk observed in human cancers, including estrogen-induced amplification of PI3K signaling.

Beyond single-gene models, combinatorial approaches have revealed the cooperative oncogenic behavior of *PIK3CA* mutations. For example, when *PIK3CA* H1047R is co-expressed with *TP53* deletion, there is a marked acceleration of tumor onset, increased histologic grade, and enhanced genomic instability [108]. In colonic epithelium, models utilizing the Villin–Cre system to drive *PIK3CA* mutation alone, inducing low-penetrance dysplastic changes, but in the context of concurrent *APC* (adenomatous polyposis coli) loss or *KRAS* activation, the same mutation contributes to rapid progression toward invasive carcinoma [111]. These findings underscore the role of *PIK3CA* as both a driver and facilitator of progression, depending on the molecular landscape.

Functionally, murine tumors harboring *PIK3CA* mutations exhibit hyperactivation of the PI3K/AKT/mTOR axis, resistance to apoptosis, increased vascularization, and metabolic reprogramming, closely paralleling features seen in human tumors. Tumor-derived cell lines from these models have been used to evaluate pharmacologic inhibitors of PI3K and have proven invaluable for preclinical testing and biomarker discovery. Notably, response to PI3Kα-selective inhibitors is often contingent on the presence of co-mutations, reinforcing the importance of contextual oncogenicity [10].

In addition to in vivo models, CRISPR (clustered regularly interspaced short palindromic repeats)/Cas9 (CRISPR-associated protein 9)-based knock-in systems in human and murine epithelial organoids have allowed the introduction of specific *PIK3CA* mutations into otherwise normal tissues, enabling precise study of early transformation events. These organoid systems, particularly those derived from the colon, lung, or mammary gland, demonstrate that *PIK3CA* activation leads to increased organoid size, survival under growth factor deprivation, and activation of downstream signaling in the absence of external stimuli [112,113].

Complementing GEMMs and organoid models, xenograft and patient-derived xenograft (PDX) models bearing endogenous *PIK3CA* mutations have been critical for evaluating therapeutic responses. Tumors with canonical H1047R or E545K mutations often show PI3K pathway dependency, but also develop resistance mechanisms, such as feedback activation of HER3 or ERK signaling, which can be explored in these models [114].

Collectively, these experimental approaches have established *PIK3CA* mutations as functional oncogenic drivers in vivo and in vitro. They have also provided mechanistic insight into the biology of PIK3CA-driven cancers, offering preclinical validation for the development of PI3K inhibitors and rational drug combinations. Importantly, these models continue to inform our understanding of tumor heterogeneity, drug resistance, and the broader therapeutic vulnerabilities associated with PI3K pathway dysregulation.

## 5. Actionability of *PIK3CA* Alterations: Therapeutic Approaches and Clinical Development

The therapeutic targeting of the PIK3CA-driven PI3Kα signaling pathway represents a pivotal advancement in precision oncology, capitalizing on the dependency of multiple tumor types on aberrant PI3K signaling for survival and proliferation. The concept of actionability encompasses the ability to therapeutically exploit PIK3CA alterations through selective inhibitors, stratified patient populations, and integration of molecular diagnostics into clinical decision-making. This section provides an extensive overview of current and emerging strategies to inhibit PIK3CA-driven oncogenic signaling, including the development of PI3Kα-specific inhibitors, combination regimens, and insights from phase II-III clinical trials.

### 5.1. PI3Kα-Selective Inhibitors and Their Mechanisms of Action

Early attempts to inhibit the PI3K pathway employed pan-PI3K inhibitors, which indiscriminately targeted all class I PI3K isoforms, resulting in dose-limiting toxicities and modest efficacy [115]. The advent of PIK3CA-selective inhibitors revolutionized the field by providing greater specificity, thereby reducing off-target effects and improving tolerability. The most clinically advanced agent in this class is Alpelisib (BYL719), an oral small molecule inhibitor that binds the ATP-binding pocket of the p110α catalytic subunit, selectively inhibiting its kinase activity. By blocking PI3Kα, Alpelisib abrogates downstream AKT/mTOR signaling, inducing cell cycle arrest and apoptosis in *PIK3CA*-mutant tumor cells [116].

### 5.2. Clinical Trials and Regulatory Approvals

The clinical development of PIK3CA-targeted inhibitors has been driven by evaluation across multiple tumor types, with the most compelling evidence emerging in HR+/HER2-negative advanced breast cancer, where *PIK3CA* mutations are highly prevalent.

However, the translation of the preclinical rationale into clinically meaningful benefit has been uneven. Across tumor types, many early pan-PI3K and isoform-selective strategies produced modest response rates, short PFS, or negative randomized results, often accompanied by dose-limiting toxicities [117,118,119,120,121,122,123]. Thus, pathway activation or the presence of a *PIK3CA* alteration should not be assumed to confer uniform PI3K dependence in patients.

A summary of the major phase II–III clinical trials evaluating PIK3CA-targeted inhibitors across different malignancies is provided in Table 2 [43,117,118,119,120,121,122,123,124,125,126,127,128,129,130,131,132,133,134,135,136,137]. Among selective PI3Kα inhibitors, alpelisib, serabelisib, and inavolisib represent successive stages in the clinical development of this therapeutic class. Alpelisib (BYL719) entered first-in-human clinical investigation in 2010 and subsequently became the first PI3Kα-selective inhibitor to achieve regulatory approval, receiving FDA approval in 2019 for patients with *PIK3CA*-mutated, HR-positive/HER2-negative advanced breast cancer. Serabelisib (TAK-117; MLN1117/INK1117) was developed during the same early generation of isoform-selective PI3Kα inhibitors; its first-in-human study established clinical feasibility but also highlighted the limited activity of monotherapy [124]. Importantly, serabelisib subsequently entered randomized phase II evaluation in combination with the dual mTORC1/2 inhibitor sapanisertib. In advanced clear-cell renal cell carcinoma, sapanisertib plus serabelisib did not improve PFS versus everolimus and was less tolerable [125]; similarly, the serabelisib-containing arm of a randomized phase II study in advanced or recurrent endometrial cancer was closed after a prespecified futility analysis [126]. Inavolisib (GDC-0077) represents a more recent generation of PI3Kα-targeted agents and is distinguished mechanistically by its ability not only to inhibit PI3Kα activity but also to promote degradation of mutant p110α. Its development culminated in the phase III INAVO120 trial, in which inavolisib plus palbociclib and fulvestrant significantly prolonged PFS in endocrine-resistant *PIK3CA*-mutated, HR-positive/HER2-negative advanced breast cancer, supporting regulatory approval in 2024 [127]. 

The pivotal Phase III SOLAR-1 trial evaluated Alpelisib combined with fulvestrant versus placebo plus fulvestrant in patients with HR+, HER2-negative advanced breast cancer harboring *PIK3CA* mutations. This study demonstrated a statistically significant improvement in PFS, leading to regulatory approval by the FDA and EMA in 2019 [43]. Nevertheless, this success should not be generalized to PIK3CA-mutant cancers as a whole. The overall clinical performance of PI3K inhibition has been considerably less impressive than the extensive preclinical evidence of pathway dependence might have suggested. In SOLAR-1, the absolute median PFS gain was clinically meaningful but still limited (11.0 vs. 5.7 months), and treatment was associated with substantial on-target toxicity: grade 3/4 hyperglycemia occurred in 36.6% of patients receiving alpelisib versus 0.7% with placebo, grade 3 rash in 9.9% versus 0.3%, and grade 3 diarrhea in 6.7% versus 0.3%; adverse events led to treatment discontinuation in 25.0% versus 4.2%, respectively [43]. These toxicities are clinically relevant because hyperglycemia and rash often emerge early, require active monitoring and supportive treatment, and may necessitate treatment interruption or dose reduction; consequently, maintaining adequate dose intensity can be challenging in routine practice. The experience with taselisib further illustrates this translational gap: in SANDPIPER, *PIK3CA* selection enriched for benefit, but the median PFS improvement was only 2 months (7.4 vs. 5.4 months), whereas serious adverse events occurred in 32.0% of taselisib-treated patients versus 8.9% with placebo; dose reductions were required in 36.5% versus 2.3%, and treatment discontinuation because of adverse events that occurred in 16.8% versus 2.3%, respectively [128]. Thus, even when pathway inhibition produces statistically significant antitumor activity, the magnitude of benefit may be partly offset by a narrow therapeutic window and the need for dose modification. More importantly, *PIK3CA* mutation has not behaved as a tumor-agnostic predictive biomarker. In the biomarker-selected NCI-MATCH cohort, taselisib produced no objective responses across 61 patients with *PIK3CA*-mutant non-breast/non-squamous-lung solid tumors, with a median PFS of only 3.1 months [129]. Similarly, mutation-selected squamous NSCLC showed minimal activity and early trial closure for futility [130], while a recent biomarker-defined HNSCC study found comparable disease control in *PIK3CA*-mutant and wild-type cohorts, arguing against clear enrichment by mutation status [131]. Conversely, the activity of broad PI3K/mTOR blockade in *PIK3CA*-wild-type HR+/HER2-negative breast cancer in VIKTORIA-1 further indicates that therapeutic dependence on this pathway cannot be inferred solely from *PIK3CA* genotype [132]. These discrepancies likely reflect several layers of biological complexity that are incompletely captured by preclinical models, including tumor-lineage-specific signaling, functional differences among *PIK3CA* variants, clonality, feedback reactivation of receptor tyrosine kinase and MAPK signaling, and co-alterations in *PTEN*, *KRAS*, *BRAF*, *ESR1*, or other pathway components [115,129]. Intrinsic resistance may therefore arise when *PIK3CA* mutation is not the dominant oncogenic dependency or when parallel signaling pathways maintain downstream survival despite PI3K blockade. Acquired resistance can subsequently emerge through adaptive relief of negative-feedback loops, reactivation of receptor tyrosine kinase signaling, restoration of AKT/mTOR output, activation of the RAS/MAPK axis, or selection of resistant subclones [115]. These adaptive responses help explain why deep and durable pathway suppression is difficult to achieve with PI3K inhibition alone and provide a biological rationale for combination strategies, although such combinations may further compound toxicity. In addition, drug selectivity, achievable target inhibition, metabolic toxicity, prior treatment exposure, and intratumoral heterogeneity can restrict the therapeutic window in patients. Taken together, these limitations are particularly evident outside HR+/HER2-negative breast cancer, where objective responses have generally been uncommon and randomized studies have frequently failed to demonstrate clinically meaningful benefit despite biological or molecular selection [117,118,119,120,121,122,123,128,129,130,131]. Accordingly, *PIK3CA* mutation is best regarded as a validated predictive biomarker only in specific drug–disease-treatment contexts, most clearly for PI3K-directed therapy combined with endocrine treatment in HR+/HER2-negative advanced breast cancer, rather than as a universal predictor of sensitivity to PI3K inhibitors. Dynamic biomarkers may further refine patient selection: in alpelisib-treated advanced breast cancer, early decreases in mutant ctDNA (circulating tumor DNA) were associated with response, clinical benefit, and improved PFS [133]. These observations support integrating *PIK3CA* genotype with tumor lineage, mutation subtype, co-genomic context, and longitudinal molecular assessment rather than using mutation status as an isolated binary biomarker.

### 5.3. Combination Therapies and Overcoming Resistance

A major challenge in the clinical application of PIK3CA inhibitors is the frequent emergence of resistance, which can be either intrinsic or acquired during treatment. These resistance mechanisms often involve reactivation of the PI3K/AKT/mTOR axis or activation of compensatory signaling networks that bypass PI3K inhibition. To overcome this limitation, several rational combination strategies have been developed with the aim of enhancing antitumor efficacy and delaying or preventing resistance. One of the most successful therapeutic strategies has been the combination of PIK3CA-targeted PI3K inhibitors with endocrine therapy in HR+ breast cancer, as previously demonstrated in the pivotal SOLAR-1 trial [43]. In this setting, dual targeting of the estrogen receptor (ER) and the PI3K pathway—exemplified by the clinically approved combination of alpelisib and fulvestrant—has produced significant clinical benefit, capitalizing on the well-established bidirectional crosstalk between ER and PI3K signaling.

Another promising therapeutic strategy involves the co-inhibition of PI3K and mTOR, a key downstream effector of the PI3K signaling pathway. By simultaneously targeting both nodes of the pathway, these combinations are designed to suppress compensatory feedback loops, prevent pathway reactivation, and achieve more profound and durable pathway inhibition. Gedatolisib, a reversible, ATP-competitive dual PI3K/mTOR inhibitor targeting all class I PI3K isoforms, exemplifies this therapeutic approach and has emerged as one of the most clinically advanced agents in this class [132]. Similarly, the concurrent use of PIK3CA inhibitors with CDK4/6 (cyclin-dependent kinase 4/6) inhibitors is being actively explored, particularly in HR+ breast cancers, based on the rationale that both pathways converge on cell cycle progression [132]. Emerging data also point to the potential of combining PIK3CA inhibitors with immunotherapy. As previously discussed, inhibition of PI3K can modulate the TME—such as by reducing the suppressive activity of regulatory T cells or altering myeloid cell function—thereby possibly enhancing the efficacy of immune checkpoint inhibitors. The combination of PI3K inhibition with immune checkpoint blockade represents one of the most promising and rapidly emerging frontiers in precision oncology. This therapeutic strategy is supported by compelling preclinical evidence demonstrating that inhibition of the PI3K pathway can reshape the tumor immune microenvironment, enhance antitumor immunity, and potentially overcome both primary and acquired resistance to immune checkpoint inhibitors. Translating these findings into the clinical setting, the ongoing phase Ib SELENA trial (ClinicalTrials.gov identifier: NCT06545682) is evaluating the combination of the PI3Kα inhibitor alpelisib with the anti-PD-1 antibody pembrolizumab in patients with metastatic breast cancer or melanoma. The primary objective is to establish the maximum tolerated dose (MTD) and recommended phase II dose (RP2D), while secondary and exploratory endpoints include preliminary antitumor activity, pharmacokinetics, and comprehensive immune biomarker analyses.

Importantly, resistance to PI3Kα inhibitors is not only driven by adaptive signaling but also by genomic alterations. Secondary mutations within *PIK3CA* itself, activation of parallel oncogenic pathways such as RAS/MAPK, or loss of tumor suppressors like *PTEN* have all been implicated in mediating drug escape. To address these complex dynamics, modern clinical trials are increasingly adopting adaptive designs that incorporate molecular re-biopsies and serial analysis of ctDNA [139,140,141]. These approaches enable real-time tracking of tumor evolution and may guide the timely adaptation of therapeutic strategies, ushering in a more personalized and responsive model of cancer treatment.

Mechanistically, resistance to PI3Kα inhibition is highly heterogeneous and can arise through both adaptive, non-genetic signaling responses and selection of resistant genomic clones. A recurrent early adaptive event is relief of negative feedback on receptor tyrosine kinases (RTKs): suppression of PI3K–AKT signaling can increase the expression and/or activity of insulin receptor (IR), insulin-like growth factor 1 receptor (IGF-1R), HER2 and HER3, thereby restoring upstream signaling and promoting reactivation of AKT and parallel MAPK/ERK pathways [142,143]. HER3 is particularly relevant because its multiple p85-binding motifs provide a powerful route for re-engaging PI3K signaling; accordingly, enhanced HER-family signaling after PI3K inhibition has been demonstrated in experimental breast cancer models, supporting combined PI3K–HER2/HER3 or PI3K–MEK strategies in selected molecular contexts [142,143]. These feedback responses illustrate why apparently complete pharmacologic inhibition of PI3Kα does not necessarily translate into durable pathway suppression at the cellular level. Genomic evolution can reinforce or bypass these adaptive responses. Acquired PTEN loss is among the best documented mechanisms of clinical resistance to PI3Kα-selective therapy. In a seminal analysis of a patient with *PIK3CA*-mutant metastatic breast cancer treated with alpelisib (BYL719), multiple progressing metastatic lesions independently acquired genetic events converging on *PTEN* loss; experimentally, *PTEN* loss shifted signaling toward PI3Kβ dependence and reduced sensitivity to PI3Kα inhibition [144]. Longitudinal tumor and plasma analyses have subsequently confirmed *PTEN* alterations as recurrent resistance events and have also identified expansion of activating *ESR1* mutations during alpelisib plus endocrine therapy, emphasizing that resistance may arise either from reactivation of the PI3K pathway or from escape through the estrogen–receptor axis [145]. Additional on-target mechanisms have now been described: serial liquid-biopsy and autopsy studies identified secondary *PIK3CA* mutations affecting the orthosteric inhibitor-binding pocket, together with activating *AKT1* alterations and other PI3K-pathway changes, in tumors progressing on PI3Kα inhibitors [146]. These observations support repeated molecular profiling at progression rather than assuming that the pretreatment *PIK3CA* genotype remains the dominant therapeutic dependency.

Downstream pathway reactivation and metabolic plasticity provide further layers of resistance. Persistent or reacquired AKT–mTORC1 signaling can maintain protein synthesis, survival and anabolic metabolism despite proximal PI3Kα blockade, while alternative effectors such as PIM (Proviral Integration site for Moloney murine leukemia virus) kinases can sustain downstream signaling in an AKT-independent manner [147]. More recently, experimental models of alpelisib-resistant *PIK3CA*-mutant breast cancer have linked constitutive mTORC1 activation to suppression of autophagy and extensive metabolic rewiring; although this phenotype confers drug resistance, it may simultaneously create vulnerabilities to agents that perturb glycolysis or mitochondrial metabolism [148]. Such metabolic approaches remain predominantly preclinical and require prospective validation, but they broaden the concept of resistance beyond purely genomic escape and suggest that metabolic state may become a therapeutically exploitable component of treatment adaptation.

Current combination strategies therefore attempt to suppress several resistance routes simultaneously. Clinically validated examples include sustained cotargeting of ER and PI3Kα, while newer approaches combine PI3K-pathway inhibition with CDK4/6 blockade or target downstream AKT/mTOR signaling. The phase III INAVO120 trial provides an important proof of principle: in patients with PIK3CA-mutated, HR-positive/HER2-negative advanced breast cancer recurring during or shortly after adjuvant endocrine therapy, the addition of the PI3Kα inhibitor inavolisib to palbociclib plus fulvestrant significantly prolonged progression-free survival compared with palbociclib–fulvestrant alone [127].

### 5.4. Biomarker Development and Patient Selection

Accurate molecular diagnostics are essential for the successful clinical deployment of PI3K inhibitors. Next-generation sequencing (NGS) panels enable sensitive detection of *PIK3CA* mutations in tumor tissue and liquid biopsies. Moreover, quantitative assessment of mutant allele frequency and clonality may inform prognosis and response prediction. Clinical guidelines now recommend *PIK3CA* mutation testing as a standard biomarker for therapy selection in advanced HR+ breast cancer [149]. Efforts are underway to standardize testing methodologies and to expand biomarker-driven approaches to other malignancies with *PIK3CA* alterations.

## 6. Conclusions and Future Perspectives

Over the past two decades, *PIK3CA* has emerged as one of the most frequently mutated oncogenes across solid tumors, playing a pivotal role in tumor initiation, progression, and therapeutic resistance. The clinical success of selective PI3Kα inhibitors, particularly alpelisib in hormone receptor-positive breast cancer, has established PI3Kα as a clinically actionable target. Nevertheless, the overall benefit of PI3K inhibition remains constrained by intrinsic and acquired resistance mechanisms, as well as treatment-related toxicities. In particular, hyperglycemia, an on-target adverse event resulting from the central role of PI3Kα in insulin receptor signaling and glucose homeostasis, represents a major cause of dose modification and treatment discontinuation. These limitations highlight the need for improved therapeutic strategies with greater selectivity and a more favorable therapeutic index.

Increasing evidence indicates that the biological and clinical impact of *PIK3CA* mutations is highly context-dependent. Their oncogenic potential is influenced by tissue lineage, co-occurring genomic alterations, epigenetic regulation, and metabolic state, suggesting that mutation status alone is insufficient to predict therapeutic response. Future patient stratification will therefore require integrated molecular profiling, incorporating co-mutation patterns, transcriptomic signatures, and longitudinal assessment of tumor evolution through liquid biopsy and other dynamic biomarkers. In this framework, liquid biopsy is particularly attractive because plasma ctDNA can provide a minimally invasive and repeatable source of tumor-derived genomic information. In advanced HR+/HER2-negative breast cancer, ctDNA-based *PIK3CA* testing can identify patients eligible for PI3Kα-directed therapy and may complement tissue genotyping when archival tissue is unavailable, inadequate, or potentially unrepresentative of contemporary disease [149,150]. Importantly, serial rather than single-time-point analysis may add a dynamic dimension to treatment monitoring: longitudinal tumor/plasma profiling during alpelisib-based therapy has detected the emergence or expansion of resistance-associated PTEN and ESR1 alterations [145], while decreases in PI3K-pathway-mutant ctDNA during alpelisib treatment have been associated with clinical benefit and longer progression-free survival [133]. Thus, ctDNA kinetics could potentially provide an early molecular readout of response or clonal escape and support molecularly informed treatment adaptation before unequivocal radiological progression. However, this application remains incompletely validated for routine PI3K-directed treatment adaptation, and negative plasma results require cautious interpretation because ctDNA shedding, tumor burden, metastatic site, assay sensitivity, and pre-analytical factors can substantially influence detectability [149,151]. Single-cell and spatially resolved approaches address a complementary limitation of bulk sequencing by resolving intratumoral heterogeneity and the anatomical organization of resistant clones and microenvironmental states. Single-cell analyses have demonstrated that *PIK3CA* mutations can be subclonal and spatially segregated from other actionable alterations, and that treatment can modify the frequency and topology of *PIK3CA*-mutant populations [152]. More broadly, single-cell transcriptomic profiling can identify rare drug-tolerant cell states and adaptive signaling programs that would be obscured in bulk measurements, including PI3K/AKT/mTOR pathway rewiring associated with therapeutic persistence [153]. Spatial transcriptomics can add positional information to these cellular states, potentially linking PI3K-pathway activation to immune, stromal, and metabolic niches and thereby helping to distinguish tumor-cell-intrinsic resistance from microenvironment-mediated escape. At present, however, the direct clinical evidence supporting spatial transcriptomics for selecting PI3K inhibitors or adapting treatment remains limited; its principal value in this field is currently translational and hypothesis-generating. Integration of serial ctDNA with single-cell and spatial profiling may ultimately enable a more complete, temporally and spatially resolved representation of *PIK3CA*-driven tumors, supporting biomarker refinement, mechanism-based combination therapies, and increasingly individualized therapeutic strategies.

Combination therapies are likely to represent the next major step in PI3K-targeted treatment. Clinical and preclinical studies support combining PI3K inhibitors with endocrine therapy, CDK4/6 inhibitors, MAPK pathway inhibitors, or immunotherapy to enhance efficacy and delay resistance. At the same time, the development of next-generation PI3K inhibitors—including mutant-selective, isoform-specific, covalent, and allosteric compounds—may improve both therapeutic efficacy and tolerability. A particularly relevant example is RLY-2608 (zovegalisib), a first-in-class, pan-mutant-selective allosteric PI3Kα inhibitor designed to preferentially inhibit mutant PI3Kα while sparing wild-type PI3Kα, thereby reducing the on-target metabolic liabilities associated with conventional orthosteric PI3Kα inhibition. Preclinical and early clinical proof-of-concept data showed antitumor activity across kinase- and helical-domain *PIK3CA* mutations with limited perturbation of glucose homeostasis, and also indicated activity against secondary *PIK3CA* mutations that confer resistance to orthosteric PI3Kα inhibitors [146,154]. Although these findings are promising, the clinical benefit and comparative therapeutic index of this strategy require confirmation in later-phase trials. Targeting downstream pathway nodes provides a complementary strategy. Capivasertib, an oral pan-AKT inhibitor, has demonstrated clinically meaningful activity in combination with fulvestrant in hormone receptor-positive/HER2-negative advanced breast cancer, particularly in tumors harboring *PIK3CA*, *AKT1*, or *PTEN* alterations; in the phase III CAPItello-291 trial, this approach significantly prolonged progression-free survival and subsequently led to regulatory approval in this biomarker-defined population [155]. These data support broader investigation of rational combinations and sequential strategies across the PI3K-AKT-mTOR axis, while emphasizing that pathway intensification must be balanced against overlapping toxicities and should ideally be guided by the dominant molecular mechanism of pathway activation or resistance.

Advances in functional genomics, CRISPR-based screening, patient-derived organoids, genetically engineered mouse models, and single-cell and spatial technologies will further refine our understanding of PI3K biology and accelerate the identification of predictive biomarkers and rational combination strategies.

In conclusion, *PIK3CA* exemplifies both the opportunities and challenges of precision oncology. Maximizing the clinical impact of PI3K-targeted therapies will require moving beyond static mutation-based treatment decisions toward dynamic, context-aware therapeutic approaches that integrate tumor genomics, evolutionary dynamics, and microenvironmental interactions. Such strategies have the potential to achieve more durable clinical responses and expand the benefit of PI3K-targeted therapies across multiple tumor types.

## Figures and Tables

**Table 1:** Prevalence and spectrum of *PIK3CA* alterations across human malignancies.

Cancer Type	Approximate *PIK3CA* Alteration Frequency	Type of Alteration	Frequency by Type
Endometrial (Endometrioid subtype)	~50–55%	Missense mutations	~50–55%
Breast (HR+/HER2−)	~35–45%	Missense mutations Amplifications	~35–40% ~5–8%
Breast (HER2+)	~25–30%	Missense mutations	~20–25%
Cervical (Squamous & Adeno)	~25–40%	Missense mutations	~25–35%
Ovarian (Clear Cell/Endometrioid)	~20–30%	Missense mutations Amplifications	~20–30% ~5%
Head & Neck (HNSCC, HPV+)	~20–25%	Missense mutations	~20–25%
Bladder/Urothelial Carcinoma	~15–20%	Missense mutations	~15–18%
		Amplifications	~5%
Colorectal Cancer	~15–20%	Missense mutations Amplifications (rare)	~15–18% <2%
Breast (Triple-Negative)	~8–12%	Missense mutations	~8–10%
Thyroid Cancer (FTC/PTC)	~10–15%	Missense mutations	~10–15%
Gastric Adenocarcinoma	~5–10%	Missense mutations Amplifications	~5–10% ~2–4%
Pediatric Low-Grade Gliomas	~5–10%	Missense mutations	~5–10%
Esophageal Squamous Cell Carcinoma	~5–8%	Missense mutations	~5–8%
Gliomas	~2–5%	Missense mutations	~2–5%
Prostate Cancer	~2–4%	Rearrangements (rare) Mutations Amplifications	Rare ~2–4% ~2%
Lung Adenocarcinoma (NSCLC)	~2–4%	Missense mutations	~2–4%
Hepatocellular Carcinoma (HCC)	~3–5%	Mutations Amplifications	~3–5% ~1–2%
Cholangiocarcinoma	~3–5%	Missense mutations	~3–5%
Pancreatic Adenocarcinoma	~1–3%	Mutations	Rare (<3%)
Melanoma (cutaneous)	~1–2%	Mutations	Rare
Pediatric Sarcomas	<1–2%	Rearrangements/fusions	Very rare

Methodological note. The values in Table 1 were not derived from a single harmonized cohort. They represent approximate ranges synthesized from the disease-specific literature discussed below and cross-referenced against major genomic resources with different ascertainment structures: TCGA is composed predominantly of primary-tumor specimens; MSK-IMPACT is a prospective clinical-sequencing cohort enriched for advanced/metastatic cancers; and COSMIC aggregates curated somatic-variant data from heterogeneous published series spanning different stages, histologies, specimen types, and sequencing methods. Unless a specific subtype is explicitly indicated, the estimates should therefore be regarded as pooled/heterogeneous rather than as frequencies restricted to either primary or metastatic disease. Variation among published estimates can additionally reflect whole-exome/whole-genome versus targeted next-generation sequencing, panel composition and depth, variant-calling thresholds, inclusion or exclusion of copy-number alterations, tumor purity, histologic/molecular subtype, and prior treatment exposure. Consequently, the ranges are intended to describe the approximate distribution of *PIK3CA* alterations across tumor types and should not be used for direct cross-cancer statistical comparisons. Adeno: adenocarcinoma; FTC: follicular thyroid carcinoma; HCC: hepatocellular carcinoma; HER2: human epidermal growth factor receptor 2; HNSCC: head and neck squamous cell carcinoma; HPV: human papillomavirus; HR: hormone receptor; NSCLC: non-small cell lung cancer; PTC: papillary thyroid carcinoma.

**Table 2:** Pivotal clinical studies of PIK3CA-targeted PI3K inhibitors across different solid malignancies.

First Author, Year	Phase	Molecular Selection	PI3K Inhibitor, Association and Comparator	Tumor Type and Disease Stage	Treatment Line	No. of pts	Key Efficacy Findings	Key Toxicity Findings	Additional Outcomes/Interpretation
Levy, 2014 [117]	Randomized phase II	No. Biomarker analyses exploratory; PIK3CA mutations/PTEN loss uncommon.	PX-866 (irreversible pan-class I PI3K inhibitor) + docetaxel vs. docetaxel	NSCLC; locally advanced, recurrent or metastatic	After 1–2 prior systemic regimens	95	Median PFS 2.0 vs. 2.9 mo (*p* = 0.65); ORR 6% vs. 0% (*p* = 0.40); OS 7.0 vs. 9.2 mo (*p* = 0.90). No efficacy benefit.	Grade ≥3 diarrhea 7% vs. 2%, nausea 4% vs. 0%, vomiting 7% vs. 0%; combination generally more toxic.	Primary endpoint: PFS. Negative study; absence of molecular selection likely relevant.
Jimeno, 2015 [118]	Randomized phase II	No. PIK3CA mutations/PTEN loss assessed retrospectively and uncommon.	PX-866 + docetaxel vs. docetaxel	HNSCC; locally advanced, recurrent or metastatic	After 1–2 prior systemic regimens	85	ORR 14% vs. 5% (*p* = 0.13); median PFS 92 vs. 82 d (*p* = 0.42); OS 263 vs. 195 d (*p* = 0.62). No significant benefit.	More grade ≥3 diarrhea (17% vs. 2%), nausea (7% vs. 0%) and febrile neutropenia (21% vs. 5%) with PX-866; anemia higher in control.	Primary endpoint: PFS. Negative, non-biomarker-selected trial.
Jimeno, 2015 [119]	Randomized phase II	No. PIK3CA mutations found in 17% of assessable tumors; PTEN loss uncommon.	PX-866 + cetuximab vs. cetuximab	HNSCC; recurrent or metastatic	After 1–2 prior systemic regimens	83	ORR 10% vs. 7%; median PFS 80 d in both arms; OS 211 vs. 256 d. No benefit by HPV status.	Higher all-grade nausea, vomiting, fatigue, diarrhea and hypokalemia with PX-866; grade ≥3 AEs more frequent without a specific pattern.	57% of assessable tumors HPV-positive. No biomarker-defined benefit.
Vansteenkiste, 2015 [120]	Open-label, two-stage phase II	Yes: PI3K-pathway activation by prescreening (PIK3CA mutation and/or PTEN alteration, per protocol); not restricted to specific PIK3CA hotspots.	Buparlisib 100 mg/day monotherapy	Metastatic squamous or nonsquamous NSCLC	Relapsed after prior systemic therapy	63	12-week PFS rate 23.3% and 20.0%, respectively; prespecified futility threshold not met and stage 2 not opened.	Known class toxicities; tolerability contributed to limited development, although efficacy futility was decisive.	Only 13.5% of 1242 prescreened patients were pathway-positive. ctDNA alterations better matched metastatic than primary tissue.
Pitz, 2015 [121]	Single-arm phase II	No mandatory selection. PTEN, PIK3CA, PIK3R1 and EGFRvIII explored retrospectively.	PX-866 8 mg/day monotherapy	Glioblastoma at first recurrence	First recurrence	33	ORR 3% (1 PR); SD 24%, median SD 6.3 mo; 6-month PFS 17%; primary endpoint not met.	Six discontinuations for toxicity; liver-enzyme elevations, diarrhea, venous thromboembolism, fatigue, nausea/vomiting and lymphopenia.	No significant biomarker–outcome association; 21% achieved durable SD.
Bowles, 2016 [122]	Randomized phase II	No PIK3CA selection; required KRAS codon 12/13 wild type. PIK3CA mutations/PTEN loss uncommon.	PX-866 + cetuximab vs. cetuximab	Metastatic colorectal cancer; anti-EGFR naïve	After both oxaliplatin and irinotecan	85	Median PFS 59 vs. 104 d (*p* = 0.77); OS 266 vs. 333 d (*p* = 0.83); no ORR benefit. Combination potentially detrimental.	Higher nausea (66% vs. 37%), vomiting (50% vs. 29%), diarrhea (64% vs. 18%), rash (66% vs. 37%); grade 3 diarrhea 19% vs. 0%.	Primary endpoint: PFS. Demonstrated lack of benefit of unselected pan-PI3K/EGFR dual blockade.
Dickler, 2018 [123]	Single-arm, open-label phase II	No mandatory PIK3CA selection; central testing when tissue available. Mutated tumors included hotspot alterations, predominantly helical/kinase-domain variants.	Taselisib 6 mg/day + fulvestrant	HR-positive/HER2-negative locally advanced or metastatic breast cancer	Postmenopausal; prior endocrine therapy allowed/expected	60	In measurable disease: confirmed ORR 38.5% in PIK3CA-mutant vs. 14.3% mutation-not-detected; CBR 38.5% vs. 23.8%.	All patients had ≥1 AE; 50% grade ≥3; 31.7% experienced serious AEs.	Activity observed irrespective of mutation status but numerically enriched in PIK3CA-mutant disease.
André, 2019 [43]	Randomized, double-blind phase III	Two prospectively defined cohorts by tumor-tissue PIK3CA status. Mutant cohort based on prespecified hotspot mutations detected by central assay (including canonical exons 7, 9 and 20 variants).	Alpelisib 300 mg/day + fulvestrant vs. placebo + fulvestrant	HR-positive/HER2-negative advanced breast cancer	After prior endocrine therapy	572; 341 PIK3CA-mutant	PIK3CA-mutant cohort: median PFS 11.0 vs. 5.7 mo; HR 0.65 (95% CI 0.50–0.85; *p* < 0.001). ORR in measurable mutant disease 35.7% vs. 16.2%.	Grade 3/4 hyperglycemia 36.6% vs. 0.7%; rash 9.9% vs. 0.3%; grade 3 diarrhea 6.7% vs. 0.3%; discontinuation 25.0% vs. 4.2%.	Established predictive clinical utility of PIK3CA mutation and led to regulatory approval.
Hotte, 2019 [138]	Two-part phase II	No PIK3CA selection. Biological rationale centered on PTEN loss; archival PTEN explored.	Part A: PX-866 monotherapy; Part B: PX-866 + abiraterone acetate	Recurrent/metastatic castration-resistant prostate cancer	Advanced CRPC; Part B included patients progressing biochemically on abiraterone	68	12-week non-progression: 33% monotherapy and 24% combination; 2 PRs and 1 PSA response in part A; no objective/PSA responses in part B.	Common: diarrhea (53/68), nausea (36), anorexia (24), fatigue (22), vomiting (20).	No correlation with PTEN status; adding abiraterone did not reverse resistance.
Saura, 2019 [134]	Randomized, double-blind, placebo-controlled phase II (neoadjuvant)	PIK3CA genotyping required but enrollment not restricted. Co-primary analyses in ITT and PIK3CA-mutant subgroup.	Taselisib 4 mg (5 d on/2 d off) + letrozole vs. placebo + letrozole for 16 wk	ER-positive/HER2-negative stage I–III operable breast cancer	Neoadjuvant first treatment	334	MRI ORR 50% vs. 39% (*p* = 0.049); in PIK3CA-mutant tumors 56% vs. 38% (*p* = 0.033). No significant pCR improvement.	Grade 3/4 GI disorders 8%, infections 5%, skin/subcutaneous disorders 5% with taselisib; serious AEs more frequent.	Biological activity enriched in PIK3CA-mutant subset, but no meaningful pCR signal.
Langer, 2019 [130]	Biomarker-driven single-arm phase II	Yes: tumor PIK3CA alteration by NGS. Primary analysis focused on substitution mutations considered potentially sensitizing; breast and non-squamous contexts not applicable.	Taselisib 4 mg/day monotherapy	Stage IV squamous NSCLC	After ≥1 platinum-based line	26	ORR 5% (1 responder); median PFS 2.9 mo; OS 5.9 mo; closed for futility.	Two possibly treatment-related deaths; one grade 4 and 11 grade 3 AEs.	Catalogued broad PIK3CA variant diversity; alteration alone insufficient for efficacy.
Dent, 2021 [128]	Randomized, double-blind phase III	Yes: centrally confirmed PIK3CA-mutant cohort using cobas PIK3CA assay; non-mutant patients enrolled separately.	Taselisib 4 mg + fulvestrant vs. placebo + fulvestrant	ER-positive/HER2-negative locally advanced or metastatic breast cancer	Progression during/after aromatase inhibitor	516 mutant ITT; 629 safety population	Median PFS 7.4 vs. 5.4 mo; HR 0.70 (*p* = 0.0037); secondary efficacy endpoints favored taselisib.	Serious AEs 32.0% vs. 8.9%; discontinuation 16.8% vs. 2.3%; dose reduction 36.5% vs. 2.3%.	Met primary endpoint, but modest benefit and poor tolerability precluded clinical utility.
Savas, 2022 [133]	Single-arm, multicohort phase II	Yes: advanced PI3K-pathway-mutant tumors; ER+/HER2− cohort mainly PIK3CA-mutant; TNBC cohort also included pathway alterations.	Alpelisib monotherapy	Heavily pretreated advanced ER+/HER2− breast cancer and TNBC	Later-line treatment	43	ER+ ITT cohort: ORR 30%, CBR 36%. Decline in mutant ctDNA at week 8 associated with response/CB and improved PFS (HR 0.24).	Expected alpelisib toxicities, particularly hyperglycemia, rash, diarrhea and fatigue; manageable with monitoring/dose modification.	Baseline ESR1 mutation associated with benefit; supports serial ctDNA as pharmacodynamic/predictive biomarker.
Krop, 2022 [129]	Single-arm phase II basket trial	Yes: activating PIK3CA mutation. Excluded breast and squamous lung cancers and tumors with KRAS or PTEN mutations. Mutations: helical 67%, kinase 18%, other 15%.	Taselisib 4 mg/day monotherapy	Advanced PIK3CA-mutated solid tumors (multiple histologies)	Heavily pretreated/no standard options	61	No CR/PR; 6-month PFS 19.9%; median PFS 3.1 mo; median OS 7.2 mo.	Most common: fatigue, diarrhea, nausea and hyperglycemia; mostly grade 1–2.	PIK3CA mutation alone was insufficient as a pan-tumor predictive biomarker.
Choueiri, 2022 [125]	Randomized phase II	No mandatory PIK3CA selection; advanced ccRCC after VEGF-targeted therapy.	Sapanisertib + serabelisib (TAK-117) vs. sapanisertib vs. everolimus	Advanced clear-cell renal cell carcinoma	After progression on/after VEGF-targeted therapy	95 total; 31 combination	Median PFS: 3.1 mo with sapanisertib + TAK-117 vs. 3.8 mo with everolimus (HR 1.37, 95% CI 0.75–2.52); ORR 7.1% vs. 16.7%. No efficacy improvement.	Grade ≥3 TEAEs 74.2% with combination; hyperglycemia and anemia 12.9% each; discontinuation for TEAEs 29.0%.	Negative phase II study; dual mTORC1/2–PI3Kα blockade was less tolerable and did not improve outcomes vs. everolimus.
Han, 2023 [126]	Randomized phase II	No mandatory PIK3CA selection; advanced/recurrent/persistent endometrial cancer.	Sapanisertib + serabelisib (TAK-117); other arms: sapanisertib, paclitaxel, or sapanisertib + paclitaxel	Advanced, recurrent or persistent endometrial cancer	After 1–2 prior regimens	234 treated; 20 combination	Sapanisertib + TAK-117 arm closed after futility analysis; no efficacy signal supporting further enrollment.	Combination: grade ≥3 TEAEs 70.0%; nausea 80%, vomiting 75%, diarrhea 65% and asthenia 50% (any grade); 6/20 discontinued because of TEAEs.	Serabelisib-containing arm failed futility assessment, underscoring limited activity and tolerability of broad vertical PI3K/mTOR pathway blockade.
Rugo, 2024 [135]	Multicenter, open-label, non-comparative phase II	Yes: centrally confirmed tumor PIK3CA mutation; prespecified activating variants accepted.	Alpelisib 300 mg/day + fulvestrant	HR-positive/HER2-negative advanced breast cancer	Immediately after progression on CDK4/6 inhibitor + aromatase inhibitor; ≤2 prior anticancer regimens	127	53.8% alive without progression at 6 mo; demonstrated activity in post-CDK4/6 setting.	Grade ≥3 hyperglycemia 29%, rash 10%, maculopapular rash 9%; serious AEs 29%; no treatment-related deaths.	Prospective evidence supporting alpelisib after CDK4/6 inhibitor exposure.
Turner, 2024 [127]	Randomized, double-blind phase III (INAVO120)	Yes: centrally confirmed PIK3CA-mutated disease; HR-positive/HER2-negative endocrine-resistant advanced breast cancer.	Inavolisib 9 mg/day + palbociclib + fulvestrant vs. placebo + palbociclib + fulvestrant	PIK3CA-mutated HR-positive/HER2-negative locally advanced or metastatic breast cancer	First-line for advanced disease after relapse during/within 12 mo of adjuvant endocrine therapy	325 (161 vs. 164)	Median PFS 15.0 vs. 7.3 mo; HR 0.43 (95% CI 0.32–0.59; *p* < 0.001). ORR 58.4% vs. 25.0%.	Grade 3/4 neutropenia 80.2% vs. 78.4%; hyperglycemia 5.6% vs. 0%; stomatitis/mucosal inflammation 5.6% vs. 0%; diarrhea 3.7% vs. 0%; discontinuation 6.8% vs. 0.6%.	Pivotal positive trial establishing clinically meaningful benefit of mutant-selective PI3Kα inhibition/degradation and supporting 2024 regulatory approval.
Konstantinopoulos, 2025 [136]	Open-label randomized phase III	No PIK3CA selection. Required no germline or known somatic BRCA mutation; biomarker analyses exploratory.	Alpelisib 200 mg/day + olaparib 200 mg BID vs. physician’s-choice paclitaxel or PLD	Platinum-resistant/refractory high-grade serous ovarian cancer	1–3 prior systemic therapies; prior bevacizumab required unless contraindicated	358	Median PFS 3.6 vs. 3.9 mo; HR 1.14; ORR 15.6% vs. 13.5%; OS 10.0 vs. 10.6 mo. Primary endpoint not met.	Safety consistent with known profiles of alpelisib and olaparib; no new/unexpected signals.	Exploratory biomarker analyses identified features among occasional responders but did not rescue overall negative result.
Hurvitz, 2026 [132]	Randomized phase III	Yes—negative selection: centrally confirmed PIK3CA wild-type disease.	Gedatolisib + palbociclib + fulvestrant; gedatolisib + fulvestrant; vs. fulvestrant	HR-positive/HER2-negative advanced breast cancer	After progression during/after CDK4/6 inhibitor + aromatase inhibitor	392	Median PFS 9.3 mo triplet vs. 2.0 mo control (HR 0.24); 7.4 mo doublet (HR 0.33); both *p* < 0.001.	Grade ≥3: neutropenia 62.3% triplet/0.8% doublet; stomatitis 19.2%/12.3%; rash 4.6%/5.4%; hyperglycemia 2.3%/2.3%; low discontinuation rates.	Shows efficacy of broad PI3K/mTOR blockade specifically in PIK3CA-WT post-CDK4/6 disease.
Voorthuis, 2026 [137]	Randomized, placebo-controlled phase II	No mandatory PIK3CA selection; ctDNA exploratory for pathway alterations, tumor fraction and resistance.	Taselisib 4 mg + tamoxifen 20 mg vs. placebo + tamoxifen	Metastatic HR-positive/HER2-negative breast cancer	Endocrine-refractory, generally >2L; prior CDK4/6 inhibitor and everolimus allowed	152	Median PFS 4.8 vs. 3.2 mo; HR 0.69 (80% CI 0.49–0.98; *p* = 0.17 under prespecified α = 0.20).	Clinically significant; diarrhea 40% any grade; benefit did not outweigh tolerability.	High baseline ctDNA tumor fraction strongly associated with worse PFS and OS.
Gauduchon, 2026 [131]	Two parallel, biomarker-defined phase II cohorts	Yes: separate PIK3CA-mutant (exons 9/20) and PIK3CA-wild-type cohorts.	Buparlisib 100 mg/day monotherapy	Refractory recurrent/metastatic HNSCC	After platinum and cetuximab; 78% had ≥2 prior systemic lines	58	2-mo DCR 38.9% WT and 36.4% mutant; limited activity and no enrichment in mutant cohort.	53.4% treatment-related grade ≥3 AEs; common hyperglycemia, lymphopenia, asthenia, hyponatremia, depression and dermatitis; 2 toxic deaths.	Trial met statistical DCR endpoint but toxicity was unacceptable; mutant cohort closed early for slow accrual.

AE: Adverse event; AI: Aromatase inhibitor; BID: Twice daily; CB: Clinical benefit; CBR: Clinical benefit rate; CDK4/6: Cyclin-dependent kinase 4 and 6; CI: Confidence interval; CR: Complete response; CRC: Colorectal cancer; CRPC: Castration-resistant prostate cancer; ctDNA: Circulating tumor DNA; DCR: Disease control rate; EGFR: Epidermal growth factor receptor; ER: Estrogen receptor; GI: Gastrointestinal; GBM: Glioblastoma; HER2: Human epidermal growth factor receptor 2; HNSCC: Head and neck squamous cell carcinoma; HR: Hazard ratio (when referring to efficacy analyses); Hormone receptor (when referring to breast cancer subtype); HR+: Hormone receptor-positive; ITT: Intention-to-treat; mo: months; MRI: Magnetic resonance imaging; mTOR: Mammalian target of rapamycin; No. of pts: number of patients; NGS: Next-generation sequencing; NSCLC: Non-small cell lung cancer; ORR: Objective response rate; OS: Overall survival; PD-L1: Programmed death-ligand 1; PFS: Progression-free survival; PI3K: Phosphoinositide 3-kinase; PIK3CA: Phosphatidylinositol-4,5-bisphosphate 3-kinase catalytic subunit alpha gene; PLD: Pegylated liposomal doxorubicin; PO: Per os (oral administration): PR: Partial response; PSA: Prostate-specific antigen; PTEN: Phosphatase and tensin homolog; RR: Response rate; SD: Stable disease; TNBC: Triple-negative breast cancer; WT: Wild type.

## Data Availability

No new data were created.

## Abbreviations

ABD	Adaptor-binding domain
AE	Adverse event
AI	Aromatase inhibitor
AKT	Protein kinase B
APC	Adenomatous polyposis coli
ARID1A	AT-rich interaction domain 1A
BAD	BCL2-associated agonist of cell death
BH	Breakpoint Cluster Region Homology
BID	Twice daily
BRAF	B-Raf proto-oncogene, serine/threonine kinase
C2	Conserved Region 2
CAC	Cervical adenocarcinoma
Cas9	CRISPR-associated protein 9
CB	Clinical benefit
CBR	Clinical benefit rate
CD8	Cluster of differentiation 8
CDK4/6	Cyclin-dependent kinase 4 and 6
CGC	Cancer Genomics Consortium
CI	Confidence interval
CIN	Cervical intraepithelial neoplasia
CNVs	Copy number variations
COSMIC	Catalogue Of Somatic Mutations In Cancer
CR	Complete response
CRC	Colorectal cancer
CRISPR	Clustered regularly interspaced short palindromic repeats
CRPC	Castration-resistant prostate cancer
CSCC	Cervical squamous cell carcinoma
ctDNA	Circulating tumor DNA
DCR	Disease control rate
EGFR	Epidermal growth factor receptor
EMA	European Medicines Agency
ER	Estrogen receptor
ERK	Extracellular signal-regulated kinase
ESR1	Estrogen receptor 1
FDA	Food and Drug Administration
FGFR	Fibroblast growth factor receptor
FGFR3	Fibroblast growth factor receptor 3
FOXO	Forkhead box O
FTC	Follicular thyroid carcinoma
GBM	Glioblastoma
GEMMs	Genetically engineered mouse models
GI	Gastrointestinal
GLUT1	Glucose transporter 1
GPCRs	G protein-coupled receptors
HCC	Hepatocellular carcinoma
HER2	Human epidermal growth factor receptor 2
HER3	Human epidermal growth factor receptor 3
HIF-1α	Hypoxia-inducible factor 1α
HNSCC	Head and neck squamous cell carcinoma
HPV	Human papillomavirus
HR	Hormone receptor
ICGC	International Cancer Genome Consortium
IDH	Isocitrate dehydrogenase
IFN-γ	Interferon-γ
IGF-1R	Insulin-like growth factor 1 receptor
IGF1R	Insulin-like growth factor 1 receptor
IL-6	Interleukin-6
IR	Insulin receptor
IRS	Insulin receptor substrate
iSH2	Inter-Src homology 2
ITT	Intention-to-treat
KRAS	KRAS proto-oncogene, GTPase
Ltf	Lactotransferrin
LYVE-1	Lymphatic vessel endothelial hyaluronan receptor 1
MAPK	Mitogen-activated protein kinase
MDSCs	Myeloid-derived suppressor cells
MEK	Mitogen-activated protein kinase kinase
MMPs	Matrix metalloproteinases
MMTV	Mouse mammary tumor virus
MRI	Magnetic resonance imaging
MSI	Microsatellite instability
MSK-IMPACT	Memorial Sloan Kettering–Integrated Mutation Profiling of Actionable Cancer Targets
MTD	Maximum tolerated dose
mTOR	Mechanistic target of rapamycin
mTORC1	Mechanistic target of rapamycin complex 1
MYC	MYC proto-oncogene, basic helix-loop-helix
NF-κB	Nuclear factor-κB
NGS	Next-generation sequencing
NK	Natural killer
NSCLC	Non-small cell lung cancer
ORR	Objective response rate
OS	Overall survival
p21^CIP1	Cyclin-dependent kinase inhibitor 1A
p27^KIP1	Cyclin-dependent kinase inhibitor 1B
pCR	Pathologic complete response
PD-1	Programmed cell death protein 1
PD-L1	Programmed death-ligand 1
PDK1	3-Phosphoinositide-dependent protein kinase-1
PDX	Patient-derived xenograft
PFS	Progression-free survival
PH	Pleckstrin homology
PI3K	Phosphoinositide 3-kinase
PI3Kα	Phosphoinositide 3-kinase alpha
PI3Kβ	Phosphoinositide 3-kinase beta
PIK3CA	Phosphatidylinositol-4,5-bisphosphate 3-kinase catalytic subunit alpha
PIK3R1	Phosphoinositide-3-kinase regulatory subunit 1
PIK3R2	Phosphoinositide-3-kinase regulatory subunit 2
PIK3R3	Phosphoinositide-3-kinase regulatory subunit 3
PIP2	Phosphatidylinositol-4,5-bisphosphate
PIP3	Phosphatidylinositol-3,4,5-trisphosphate
PTC	Papillary thyroid carcinoma
PTEN	Phosphatase and tensin homolog
RAF	Rapidly accelerated fibrosarcoma kinase
RAS	Rat sarcoma (small GTPase family)
RAS-GTP	Guanosine triphosphate-bound RAS
RBD	RAS-binding domain
RP2D	Recommended phase II dose
RTKs	Receptor tyrosine kinases
SH2	Src homology 2
SH3	Src homology 3
STAT3	Signal transducer and activator of transcription 3
TAMs	Tumor-associated macrophages
TCGA	The Cancer Genome Atlas
TEAEs	Treatment-emergent adverse events
TERT	Telomerase reverse transcriptase
TKIs	Tyrosine kinase inhibitors
TME	Tumor microenvironment
TNBC	Triple-negative breast cancer
TNF-α	Tumor necrosis factor-α
TP53	Tumor protein p53
Tregs	Regulatory T cells
TREM2	Triggering receptor expressed on myeloid cells 2
TSC2	Tuberous sclerosis complex 2
VEGF	Vascular endothelial growth factor
VUS	Variant of uncertain significance
WAP	Whey acidic protein
WNT	Family of secreted signaling proteins, historically named from the fusion of the Wingless (Wg) gene in Drosophila and the Int-1 gene in mice
